# Band Gap Tuneability in Antiperovskite‐Based Nitrides *AE*_3_ *Pn*N and Imides *AE*_5_ *Pn*_2_(NH)_2_ (*AE* = Ca, Sr; *Pn* = As, Sb, Bi)

**DOI:** 10.1002/anie.1423389

**Published:** 2026-04-01

**Authors:** Thanh G. Chau, Florian Wolf, Dan Han, Saloni, Teak D. Boyko, Stefan S. Rudel, Thomas Bein, Hubert Ebert, Alexander Moewes, Wolfgang Schnick

**Affiliations:** ^1^ Department of Chemistry and Center for NanoScience (CeNS) University of Munich (LMU) Munich Germany; ^2^ School of Materials Science and Engineering Jilin University Changchun China; ^3^ Department of Physics and Engineering Physics University of Saskatchewan Saskatoon Saskatchewan Canada; ^4^ Canadian Light Source Saskatoon Saskatchewan Canada

**Keywords:** ammonothermal synthesis, antiperovskite derivatives, lead‐free perovskite, photovoltaics

## Abstract

Inorganic antiperovskites with the formula *X*_3_ *A*N (*X* = Ba, Sr, Ca, Mg; *A* = As, Sb) have recently been reported to exhibit excellent optoelectronic properties including small carrier effective masses, suitable direct bandgaps, high optical absorption coefficients as well as allowed optical transitions at the band edges. Using the ammonothermal method, we have synthesized the imide antiperovskites *AE*_5_ *Pn*_2_(NH)_2_ (*AE* = Ca, Sr; *Pn* = As, Sb, Bi). The crystal structures of *AE*_5_ *Pn*_2_(NH)_2_ were solved and refined in the orthorhombic space group *Pbam* by single‐crystal x‐ray diffraction (scXRD), and further confirmed using powder X‐ray diffraction (pXRD) and Raman spectroscopy. Depending on the ion size ratio between *AE* ^2+^ and *Pn* ^3–^, different degrees of octahedral tilting can be observed. Soft X‐ray spectroscopy was used to study the band gap and electronic structure, and revealed the presence of oxygen impurities. The *AE*_5_ *Pn*_2_(NH)_2_ compounds can further react to form the ternary antiperovskites *AE*_3_ *Pn*N. Density functional theory calculations reveal favorable transport and optical properties. Narrow direct band gaps in the range of 0.87–1.76 eV could be verified experimentally, making *AE*_5_ *Pn*_2_(NH)_2_ not only suitable as precursor materials for the corresponding *AE*_3_ *Pn*N antiperovskites, but also as promising candidates for solar cell absorber materials.

## Graphical abstract

A series of imide‐based defect antiperovskites *AE*_5_ *Pn*_2_(NH)_2_ (*AE* = Ca, Sr; *Pn* = As, Sb, Bi) has been synthesized using the ammonothermal method. DFT calculations and spectroscopy revealed direct band gaps suitable for photovoltaic absorber materials. The discovered compounds can further be used as precursors for the synthesis of *AE*_3_ *Pn*N antiperovskites as showcased by high‐temperature *p*XRD measurements.

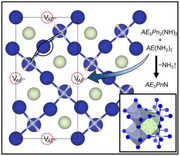

## 1 Introduction

Antiperovskites or inverse perovskites, with the general formula *X*_3_ *AB*, are derivatives of the commonly known perovskites, where the cation and anion positions are interchanged. An analogous structural diversity similar to halide, oxide, and chalcogenide perovskites is observed, which is displayed in a wide range of applications and properties such as thermoelectrics [1, 2, 3, 4], optoelectronic functionality [5], magnetism [6, 7], superconductivity [8, 9, 10], topological properties [11], ionic conductivity [12, 13], and high‐temperature ceramics [14, 15].

A growing interest in antiperovskite research can therefore be observed in both theoretical and experimental studies. Hence, several strategies to engineer the general *X*_3_ *AB* structure have been devised. For the series of *AE*_3_ *Pn*N, the optoelectronics studies were extended to the quaternary ordered *AE*_6_ *PnPn*’N_2_ antiperovskites (*AE* = Ca, Sr; *Pn* = P, As, Sb, Bi), which have been predicted to show suitable properties as photovoltaic absorber materials [5, 16, 17]. The heavier homologue materials Ca_3_SbAs [18] as well as Ca_3_SbN and Ca_3_BiN [19], also show promising thermoelectric properties. Sr_3_SbN and Sr_3_BiN crystallize in the hexagonal antiperovskite structure where the pronounced anisotropy and the anharmonic vibrations of the N atoms result in a low thermal conductivity and a favorable thermoelectric performance [20].

The Mn_3_ *A*N compounds (*A* = Cu, Zn, Ga, Fe) are transition‐metal‐based antiperovskites where the compounds show several magnetic transitions, with negative thermal expansion behavior and giant magnetocaloric effect being some of their numerous magnetic properties [6, 7, 15].

In the case of antiperovskite‐based ionic conductors, several studies can be found where the structural flexibility is exploited by the incorporation of polyanions or (rotational) anion clusters, which facilitate a high ionic conductivity due to the coupled anion rotation and ion migration [13, 21, 22, 23].

Other ways to incorporate polyanions have been shown for the nitridometallate Sr_7_Sn_3_N_2_ [24] and Sr_11_Ge_4_N_6_ [25], where antiperovskite‐type slabs are separated by the Zintl polyanion Sn_4_^8−^ or by GeN_2_^4−^. Most of the alkaline‐earth‐based antiperovskite compounds can be synthesized in bulk by solid‐state reactions starting from stoichiometric mixtures of the metals under nitrogen atmosphere [26]. Additionally, several alternative synthetic routes have been reported thus far. For Ca_3_TlN [27] and Ca_3_AuN [28], the metals were alloyed and subsequently heated under nitrogen atmosphere. The utilization of *AE*_3_N_2_ nitrides and the pnictide elements is an additionally established route where, N_2_ is formed in a redox reaction [29].

For the synthesis of Sr_7_Sn_3_N_2_ as well as the antiperovskites showing an elpasolite‐type superstructure, a sodium‐flux with NaN_3_ as a nitrogen‐source was employed [24, 30]. But to utilize antiperovskites for various applications, thin film synthesis is crucial. Several successful syntheses of antiperovskite thin films can be found for transition‐metal‐based compounds [31, 32, 33, 34].

Mg_3_SbN, in contrast, is so far the only alkaline‐earth‐based antiperovskite compound that has been successfully synthesized both in bulk [35] and as thin films.[36] Theoretical calculations, as well as electrical and optical property measurements, have shown promising results for its application as a photovoltaic absorber. In our previous work, we employed the ammonothermal method to synthesize the multinary imide‐based defect antiperovskites, *AE*_5_As*Pn*(NH)_2_ (*AE* = Ca, Sr; *Pn* = Sb, Bi) [37].

In this work, a series of antiperovskite‐related imide compounds, namely Ca_5_As_2_(NH)_2_, Ca_5_Sb_2_(NH)_2_, Ca_5_Bi_2_(NH)_2_, Sr_5_Sb_2_(NH)_2_, and Sr_5_Bi_2_(NH)_2_ was synthesized. The title compounds show direct band gaps covering a wide range of the optical spectrum (0.78–2.01 eV) making them potentially suitable as solar cell absorbers. For the synthesis of the imide‐compounds, the ammonothermal method was employed, resulting in a crystal structure consisting of square pyramidal *AE*_5_NH‐units and *AE*_10_ *Pn Zentaur*‐polyhedra with channels of vacancies along the [001] direction. The title compounds are related to the antiperovskite structure (ABX_3_) by a formal aliovalent substitution of one *AE* ^2+^‐ion with 2H^+^‐ions, which can be reversed by a further reaction with an *AE*‐source such as *AE*(NH_2_)_2_.

The crystal structures of the *AE*_5_ *Pn*_2_(NH)_2_ compounds crystallizing in space group *Pbam* (no. 55), were solved and refined from single‐crystal X‐ray diffraction (scXRD) data and verified by powder X‐ray diffraction (pXRD), energy‐dispersive X‐ray (EDX) spectroscopy, and Raman spectroscopy. High temperature‐dependent diffraction data (HTXRD) demonstrate the thermal behavior as well as the subsequent reaction to the *AE*_3_ *Pn*N compounds. Soft X‐ray emission spectroscopy (XES) and X‐ray absorption near‐edge spectroscopy (XANES) were employed to probe the electronic band gap at the N K‐edge and to reveal the presence of oxygen as reflected by the O K‐edge spectra. Density functional theory (DFT) calculations of the electronic structures and optoelectronic property measurements were performed, revealing direct band gaps, high absorption coefficients, and spectroscopically limited maximum efficiency of over 20%, rendering the compounds potentially suitable for solar cell applications.

## 2 Results and Discussion

### 2.1 Synthesis

**FIGURE 1:**
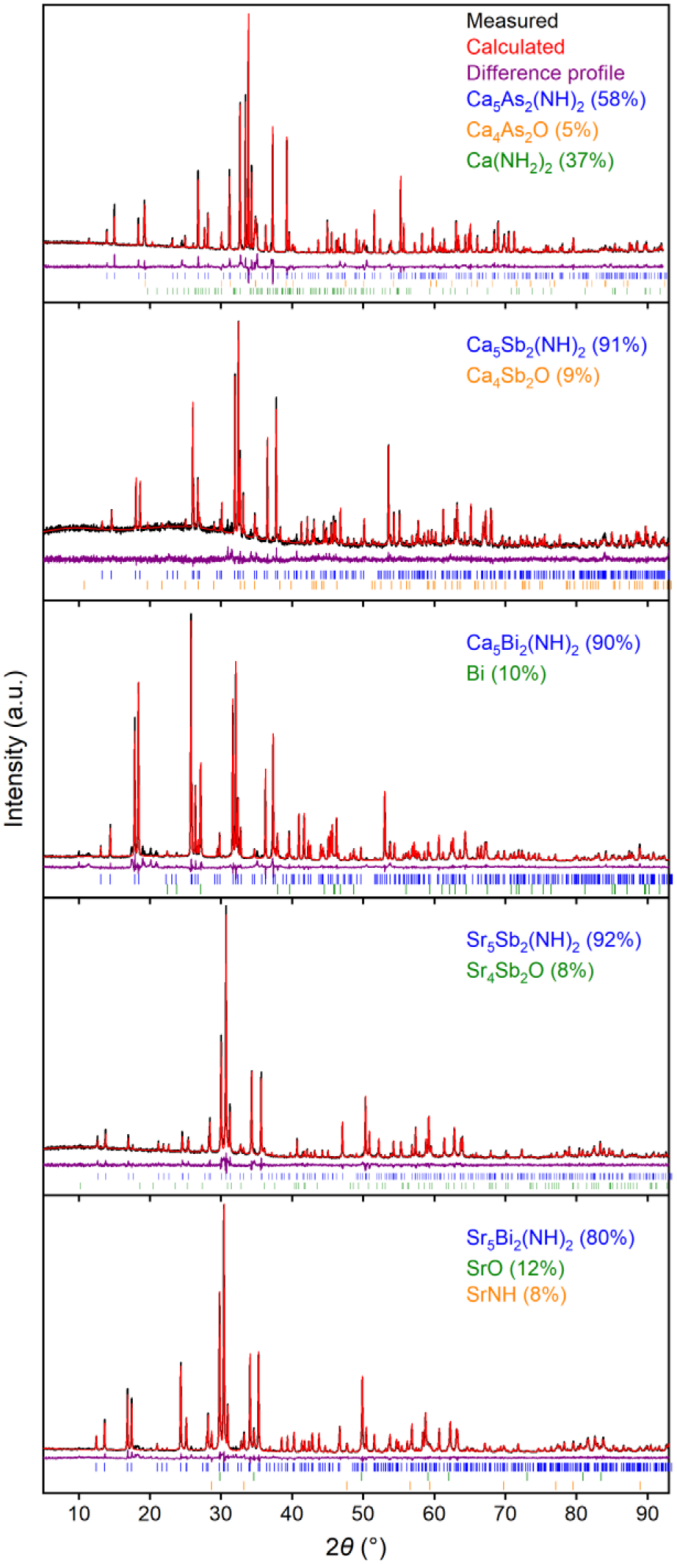
Rietveld refinement of *AE*_5_ *P* *n*_2_(NH)_2_. Black: measured data points, red: Rietveld fit, purple: difference plot, and colored bars correspond to various phases.

The title compounds were synthesized from *AE*_3_N_2_ or *AE*H_2_ and elemental *Pn* under supercritical ammonia at 1000 K and an autogenous pressure of 50–120 MPa. Custom‐made autoclaves (Haynes 282) with niobium liners were used as reaction vessels [38]. Due to the air sensitivity of the product and starting materials, all preparations were performed inside a glovebox under dry argon atmosphere. A detailed description of the synthesis details is given in the Supporting Information 1.1. An excess of the *AE*‐source was used during synthesis to ensure complete conversion of the *Pn*‐source, which resulted in the formation of *AE*(NH_2_)_2_ as a byproduct in the colder zone of the autoclave. This colorless byproduct could be easily separated and removed mechanically from the bulk material (except in the case of Ca_5_As_2_(NH)_2_). Slight inadvertent exposure to oxygen impurities during preparation also, resulted in the formation of around 5%–9% *AE*_4_ *Pn*_2_O (see Figure 1). All samples were further characterized using pXRD, scXRD, EDX‐, UV–vis‐, and Raman‐spectroscopy. The crystal structures were obtained from scXRD [39]. Soft X‐ray spectroscopy (XES and XANES) was employed at the O and N K‐edge on Ca_5_Bi_2_(NH)_2_, Ca_5_Sb_2_(NH)_2,_ and Sr_5_Sb_2_(NH)_2_ to examine the presence of oxygen (Figure S4) and to probe the electronic structure.

Detailed single‐crystal data are tabulated in the Table S1, with detailed crystallographic data for each compound in Tables S2–S11. Rietveld refinements of the pXRD data are shown in Figure 1, with detailed data in Table S12. EDX‐measurements confirmed the atomic ratio A*E*:*Pn* of 5:2 and are tabulated in the Supporting Information, Table S15. Due to the high air sensitivity of the title compounds, varying concentrations of O and N were observed in the EDX‐measurements after transfer in air and have therefore been neglected. In the case of Ca_5_As_2_(NH)_2_, slightly higher amounts of Ca were detected which can be attributed to the higher phase fraction of Ca(NH_2_)_2_. The series of *AE*_3_ *Pn*N‐compounds was synthesized from the *AE*_5_ *P* *n*_2_(NH)_2_‐compounds with additional *AE*(NH_2_)_2_ or directly from *AE*_3_N_2_ and elemental *Pn* in high‐frequency furnaces at 1250 K in an argon atmosphere. Rietveld refinements of the *AE*_3_ *Pn*N‐samples are shown in Figure S1, with detailed data for each sample in Table S13. The quaternary antiperovskites Ca_6_AsSbN_2_ and Ca_6_SbBiN_2_ show no hints for an A‐site ordering of As/Sb and Sb/Bi, and have therefore been treated as a solid solution between Ca_3_AsN‐Ca_3_SbN (Ca_3_As_1‐_ *_x_* Sb*_x_* N) and Ca_3_SbN‐Ca_3_BiN (Ca_3_Sb_1‐_ *_x_* Bi*_x_* N), where both compounds crystallize in $Pm\bar{3}m$ (no. 221), the aristotype antiperovskite structure. The resulting lattice parameter *a* and the corresponding cell volumes of these quaternary antiperovskites are in the range of the respective boundary compounds, but deviate slightly from Vegard's law (Figure S2), although a more extensive investigation of the solid solutions would be necessary to discuss this deviation.

### 2.2 Crystal Structure

**FIGURE 2:**
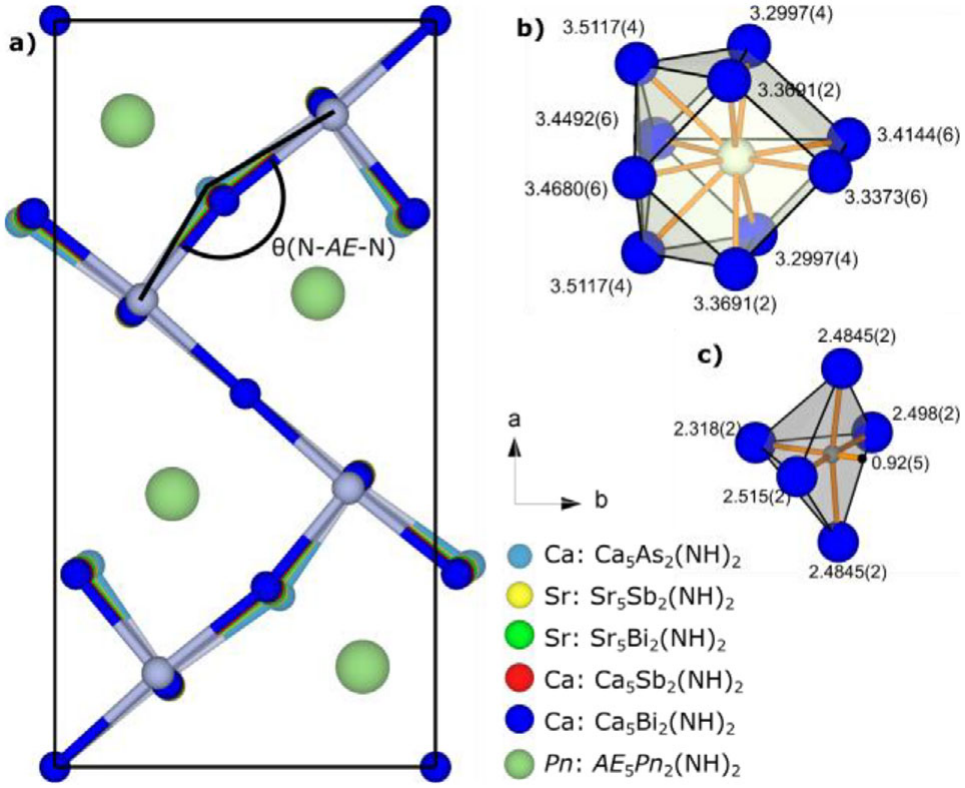
(a) *AE*_5_NH defective octahedra network of *AE*_5_ *Pn*_2_(NH)_2_, (b) *AE*_10_ *Pn* ‐coordination in Ca_5_Bi_2_(NH)_2_ (c) *AE*_5_NH‐coordination in Ca_5_Bi_2_(NH)_2_. All distances are given in Å.

The title compounds *AE*_5_ *Pn*_2_(NH)_2_ (*AE* = Ca, Sr; *Pn* = As, Sb, Bi), depicted in Figure 2a, crystallize in space group *Pbam* (no. 55) and adopt an antiperovskite‐related structure, inversely related to the Ca_2_Mn_2_O_5_ structure [40, 41]. The structure consists of *Pn*‐centered *AE*_10_ *Pn* (Zentaur) polyhedra (Figure 2b) and distorted NH‐centered *AE*_5_NH square pyramid units (Figure 2c) which are linked with adjacent *AE*_5_NH units, thus forming a defective octahedral network with channels of vacancies along the crystallographic *c*‐axis. The NH groups in these *AE*_5_NH units are pointing toward each other with N(1)‐H(1) distances of 0.74(12)‐0.97(7) Å. The distortion of the square pyramid shape manifests itself as three similarly long equatorial N‐*AE*‐distances, one significantly longer equatorial N‐*AE*‐distance and a short apical N‐*AE*‐distance, see Table S14. Similar distortions of the square pyramidal bonding situation have also been observed in the isotypic Ca_2_Mn_2_O_5_ compound [40, 41]. Further insight into the imide bonding situation can be obtained from the calculated electron density maps in Figure S6. The vibrational spectroscopy measurements (Figure 5c), also further verify the presence of NH‐bonds in our determined crystal structures. The average N‐*AE*‐distances in the *AE*_5_ *Pn*_2_(NH)_2_ compounds are all in good agreement with the literature, with 2.40–2.46 Å for N─Ca distances and 2.60–2.61 Å for N‐Sr‐distances, which is in the same range as observed in the *AE*_3_ *Pn*N‐compounds. The *AE*_5_NH‐units can therefore be seen as rigid units, similar to the *AE*_6_N‐octahedra in the *AE*_3_ *Pn*N antiperovskites. Depending on the alkaline‐earth and pnictide element, a tilting of the *AE*_5_NH‐based network can be observed, which according to the Glazer notation can be represented as *a* ^0^ *a* ^0^ *c^−^* tilt system. The degree of the network tilting can be quantified by Δ*θ*, half the deviation of the *θ*(N–*AE–*N) angle from 180°, which (with the exception of Sr_5_Bi_2_(NH)_2_) increases with decreasing Goldschmidt tolerance factor of the corresponding *AE*_3_ *Pn*N compound, see Table 1. The trend of network deformation can be assigned to the ion size ratio of the compounds as it is already done by the Goldschmidt tolerance factor. The exception of Sr_5_Bi_2_(NH)_2_ occurs most likely due to the smaller ionic radius of NH^2−^ compared to N^3^ *^−^*. Estimations of the NH^2−^ radius have been done by Roobottom et al. [42]. using the thermochemical radii (NH^2−^: 0.13(2) nm; N^3^ *^−^*: 0.18(4) nm), but its values are less representative for NH^2−^ ions due to their non‐spherical shape. Calculation attempts of the Goldschmidt factor *f* for the title compounds have therefore been omitted. The observed *Pn─AE*‐distances in the *AE*_10_ *Pn*‐(Zentaur)‐polyhedra of the title compounds are all slightly shorter compared to the corresponding *AE*_3_ *Pn*N‐compounds, which is expected as it results from the tilted polyhedral network due to the introduced *AE*‐vacancy.

**TABLE 1:** Average distances and tilt angles in Ca_5_As_2_(NH)_2_, Ca_5_Sb_2_(NH)_2_, Ca_5_Bi_2_(NH)_2_, Sr_5_Sb_2_(NH)_2,_ and Sr_5_Bi_2_(NH)_2_. Goldschmidt tolerance factor *f* and average distances of the corresponding the *AE*_3_ *Pn*N antiperovskites for comparison.

formula	Ca_5_As_2_(NH)_2_	Sr_5_Sb_2_(NH)_2_	Ca_5_Sb_2_(NH)_2_	Sr_5_Bi_2_(NH)_2_	Ca_5_Bi_2_(NH)_2_
θ(N–*AE–*N) / °	159.42	163.77	167.44	164.46	168.23
Δθ / °	10.29	8.12	6.28	7.77	5.89
$\bar{d}$(N‐*AE)* / Å	2.4057	2.5999	2.4463	2.6120	2.4602
$\bar{d}$(*Pn*‐*AE)* / Å	3.2662	3.5630	3.3695	3.5936	3.4030

formula	Ca_3_AsN^*^, ^a^	Sr_3_SbN^a^	Ca_3_SbN^b^	Sr_3_BiN^a^	Ca_3_BiN^a^
*f*	0.85	0.90	0.92	0.93	0.95
$\bar{d}$(N–*AE)* / Å	2.3993	2.5863	2.4271	2.6035	2.4442
$\bar{d}$(*Pn–AE)* / Å	3.3741	3.6575	3.4324	3.6818	3.4566

* Ca_3_AsN crystallizes in the orthorhombic space group *Pbnm* (no. 62).

^a^ Ref [43],

^b^ Ref [29]

### 2.3 Temperature Dependent Powder X‐Ray Diffraction

**FIGURE 3:**
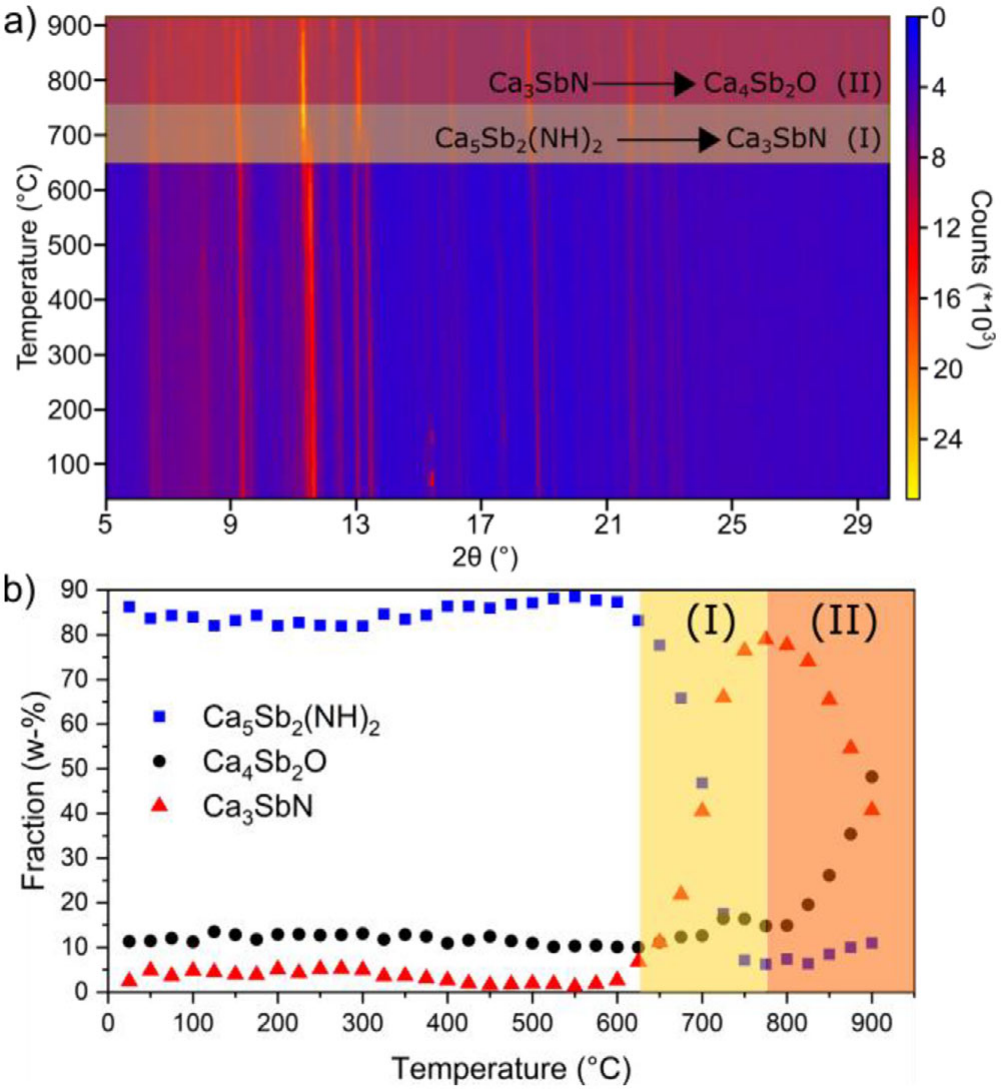
(a) Temperature‐dependent powder X‐ray diffraction (25 K steps) of Ca_5_Sb_2_(NH)_2_ and (b) refined phase fraction of Ca_5_Sb_2_(NH)_2_, Ca_4_Sb_2_O and Ca_3_SbN with decomposition of imide (I) and oxidation of nitride (II) marked.

To study temperature‐induced phase transitions and decomposition reactions, temperature‐dependent X‐ray diffraction measurements were carried out for Ca_5_Sb_2_(NH)_2_ and Ca_3_SbN (Figures 3a and S3a) in the range of 25–900 °C in steps of 25 °C. The measured Ca_5_Sb_2_(NH)_2_ samples show a small number of Ca_4_Sb_2_O‐impurities of around ∼10% phase fraction, which remained stable up to around 750 °C (Figure 3b). The low amounts of <5% Ca_3_SbN observed up to 400 °C are a result of the overlapping reflection positions at these temperatures and are therefore treated as an artifact of the refinement. A detailed analysis of the thermal behavior of Ca_5_Sb_2_(NH)_2_ and Ca_3_SbN is shown in Figure S3b. Ca_5_Sb_2_(NH)_2_ is stable up to around 600 °C with thermal expansion coefficient α in the range of 0.66‐2.41 × 10^−4^ K^−1^. The octahedral network in Ca_5_Sb_2_(NH)_2_ shows no disruption along the *c*‐axis, which results in a thermal expansion coefficient α_c_ of 0.66(1) × 10^−4^ K^−1^ similarly to Ca_3_SbN with α_a_  =  0.73(8) × 10^−4^ K^−1^ (determined by a separate HTXRD measurement). At 625 °C, the beginning decomposition of Ca_5_Sb_2_(NH)_2_ can be observed, indicated by the disappearance of a number of reflections as well as the apparent merging of reflections, due to the close symmetry relation of Ca_5_Sb_2_(NH)_2_ and Ca_3_SbN. Such a decomposition of Ca_5_Sb_2_(NH)_2_ to Ca_3_SbN can be described by equation (I).

(I) $$Ca_{5}Sb_{2}{\left(\mathrm{NH}\right)}_{2}+2\mathrm{Ca}{\left(NH_{2}\right)}_{2}\to 2Ca_{3}\mathrm{SbN}+2NH_{3}↑$$

The resulting Ca_3_SbN‐phase further reacts at around 775–800 °C to form Ca_4_Sb_2_O, for which the following equation (II) can be formulated.

(II) $$\;2Ca_{3}\mathrm{SbN}+1.5O_{2}\to Ca_{4}Sb_{2}O+N_{2}↑+2\mathrm{CaO}$$

The oxygen in Equation (II) is most likely originating from either a diffusion through the quartz‐capillary [44] or a leakage, caused by the reaction with the quartz capillary at elevated temperature. A bulk synthesis of *AE*_3_ *Pn*N from *AE*_5_ *Pn*_2_(NH)_2_ also succeeded upon heating at around 800–1000 °C in tungsten crucibles under N_2_ atmosphere.

### 2.4 K‐Edge XES and XANES Spectra

The measured and calculated N Kα XES and 1s XANES spectra of *AE*_5_ *P* *n*_2_(NH)_2_ are shown in Figure 5a–c, respectively. The N Kα XES spectra for all samples were recorded using an excitation energy of 440 eV, well above the nitrogen K‐edge binding energy. The experimental XES spectra show good agreement with the calculated spectra, indicating that the structural model is correct, electron correlation can be neglected and the main spectral features originate from the nitrogen atoms in the crystalline compounds. However, the XANES spectra are dominated by characteristic vibrational peaks of N_2_ gas, labeled *e* through *i*. All these XES and XANES measurements contain interstitial N_2_ gas features resulting from X‐ray irradiation during the experiment. Previous studies have shown that irradiating NH_3_ ice films at 20 K with 150 eV photons induces photochemical reactions that lead to N_2_ formation through intermediate radical species such as NH, NH_2_, N_2_H_4_, N_2_H_2_, and N_2_H_3_ [45]. Therefore, it is concluded that the imide (NH) groups within the *AE*_5_ *Pn*_2_(NH)_2_ samples were slightly decomposed under soft X‐ray exposure, which is a common occurrence in nitrides [46]. To account for the spectral contribution from N_2_ gas in the calculated spectra, experimentally measured N_2_ gas XES and XANES spectra (shown in blue) were added to the simulated spectra of the compounds (shown in magenta) to provide a more meaningful comparison between measured and calculated spectra.

For the *AE*_5_ *Pn*_2_(NH)_2_ samples: Ca_5_Sb_2_(NH)_2_, Ca_5_Bi_2_(NH)_2_ and Sr_5_Sb_2_(NH)_2_, 40, 50 and 40% of the N_2_ gas spectrum were added to the XES calculations and 90% for Ca_5_Sb_2_(NH)_2_, and 70% for both Ca_5_Bi_2_(NH)_2_ and Sr_5_Sb_2_(NH)_2_ to the XANES calculations. However, these values should not be interpreted as the actual fraction of the sample that has decomposed. In particular, the absorption cross‐section and fluorescence yield of N_2_ gas are significantly higher than those of the solid phase. In addition, the N_2_ formed during the experiment is trapped within the system and has much higher effective density than under ambient conditions. As a result, even a relatively small amount of decomposed material can produce a large spectral contribution of N_2_ gas. This explains high contributions of N_2_ gas in the spectra. Overall, the distinct spectral features labeled a through j in the XES and XANES spectra were well reproduced in the combined (calculated + N_2_) spectra (shown in red), confirming that the inclusion of the N_2_ gas contribution is essential for accurately reproducing the X‐ray spectral features in all three samples.

### 2.5 Band Gap Estimation From XES and XANES Spectra

**FIGURE 4:**
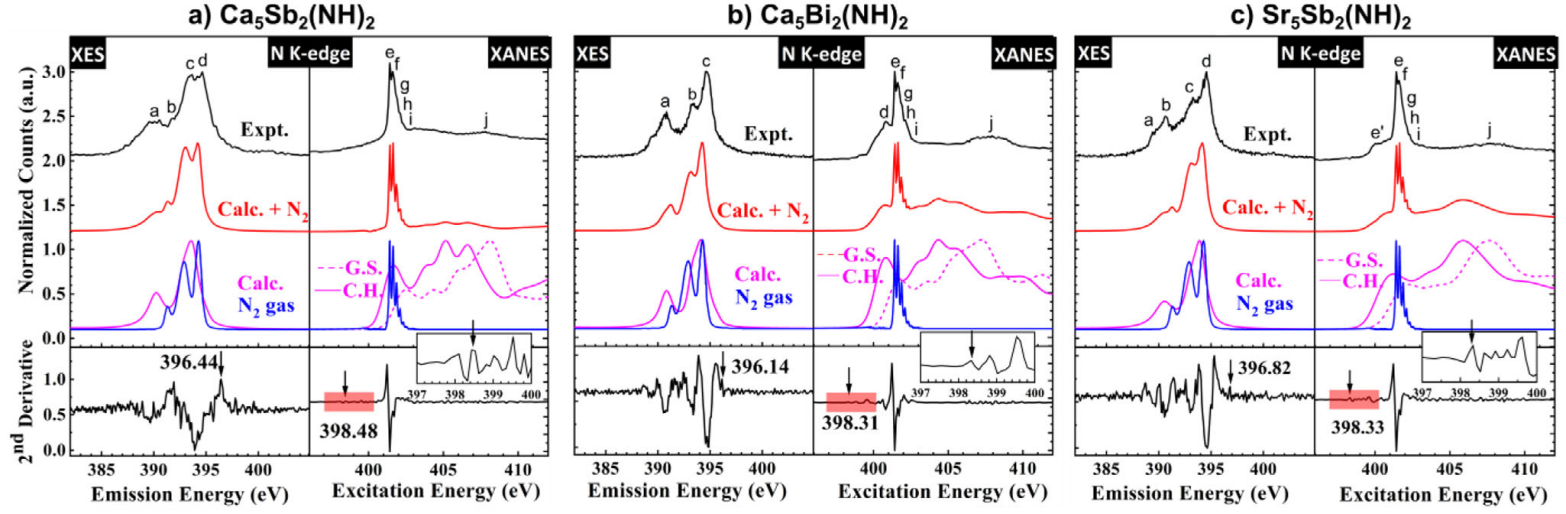
Measured (black) and calculated (magenta) N K‐edge XES and XANES spectra in PFY (Partial Fluorescence Yield) mode of (a) Ca_5_Sb_2_(NH)_2_, (b) Ca_5_Bi_2_(NH)_2_ and (c) Sr_5_Sb_2_(NH)_2_ calculated for structures without oxygen. The measured N_2_ gas spectra (blue) were added to the simulated spectra (magenta) to account for interstitial N_2_ gas contributions, resulting in the combined spectra (red). The bottom panels show the second derivatives of the XES and XANES spectra to unambiguously estimate experimentally the electronic band gaps. Inserts show the magnified area (highlighted in red).

The N Kα XES and 1s XANES spectra of *AE*_5_ *Pn*_2_(NH)_2_ were used to estimate their electronic band gaps, which represents the energy difference between the valence band maximum and the conduction band minimum (in the ground state). To determine the location of the band edges, the second derivative method was used [47, 48]. In this method, the valence band maximum and conduction band minimum are identified from the first peaks above the noise level at the upper edge of the XES and the lower edge of the XANES spectra, respectively, as shown by arrows in the bottom panels of Figures 4a–c. Compared to other techniques such as linear extrapolation, this method offers a more consistent and less ambiguous determination of the band gap. A more detailed description of the band gap estimation using the XES and XANES spectra is given in the supporting information section 1.7. The band gaps estimated from the spectral onsets for Ca_5_Bi_2_(NH)_2_, Ca_5_Sb_2_(NH)_2_, and Sr_5_Sb_2_(NH)_2_ were found to be 2.16 ± 0.40 eV, 2.04 ± 0.42 eV, and 1.51 ± 0.45 eV, respectively. After applying the core‐hole shifts of 0.05 eV for Ca_5_Bi_2_(NH)_2_, and 0.04 eV for both Ca_5_Sb_2_(NH)_2_ and Sr_5_Sb_2_(NH)_2_, the final corrected band gap values are 2.21 ± 0.45 eV, 2.08 ± 0.42 eV, and 1.55 ± 0.45 eV, respectively. The uncertainty of ± 0.4 eV associated with the band gap values was estimated by selecting different possible peak positions near the valence band maximum (VBM) and conduction band minimum (CBM) in the second derivatives of XES and XANES spectra and averaging the corresponding band gap values as well as taking into account the spectral resolution limits of monochromator, spectrometer and detector.

The calculated band gaps were determined using the experimental crystal structures obtained from the scXRD measurements. The values using the mBJ exchange‐correlation functional for Ca_5_Bi_2_(NH)_2_, Ca_5_Sb_2_(NH)_2_, and Sr_5_Sb_2_(NH)_2_ are 1.57, 1.67, and 1.68 eV, respectively. These values are significantly smaller than the experimentally measured band gaps. While generally DFT tends to significantly underestimate band gaps, the specific situation for the systems studied here is more complex. The discrepancy between experimental and calculated band gaps arises because the program package WIEN2k that was used for those calculations (see SI1.7, 1.8) defines the band gap strictly as the energy difference between the VBM and CBM without accounting for spectral weight. In contrast, the experimental band gaps were determined from the N K‐edge, where the calculated partial density of states (see Supporting Information 2.2, Figure S5) shows that the nitrogen contribution at the VBM is extremely small. Consequently, the measured spectra are dominated by transitions from states with higher N spectral weight, while the negligible contribution from the weak N states at the VBM is not detected experimentally. This results in a larger band gap in the measurements compared to the DFT calculated values.

### 2.6 Vibrational Spectroscopy

**FIGURE 5:**
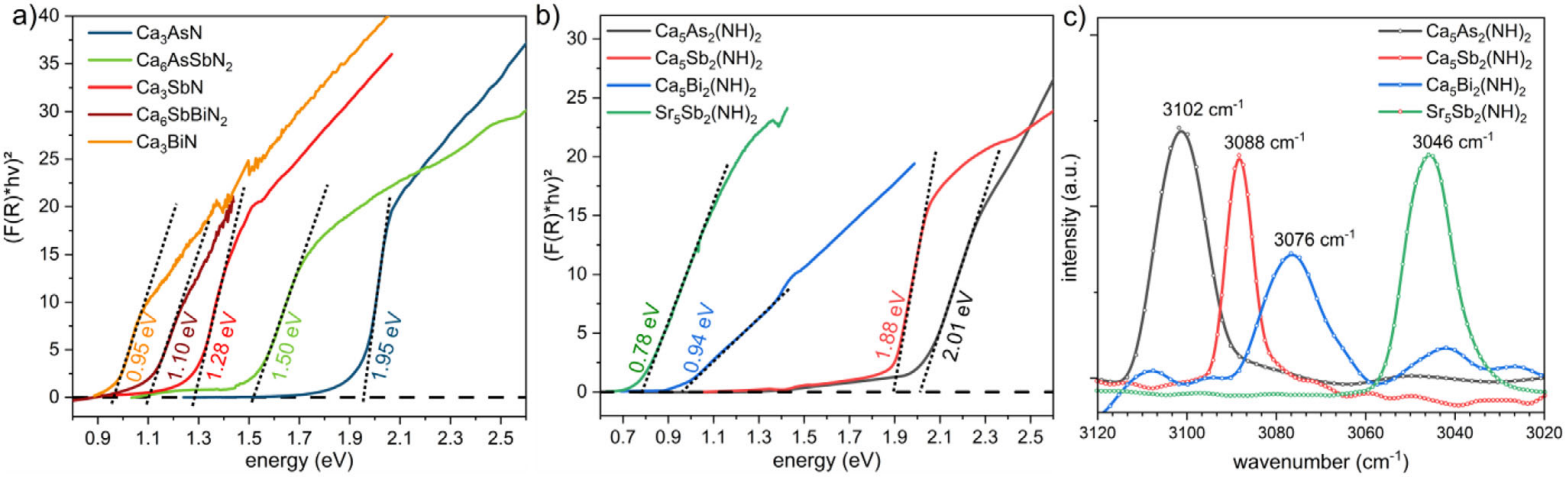
Tauc‐plots of (a) Ca_3_ *Pn*N and Ca_6_ *PnPn´*N_2_ antiperovskites and (b) *AE*_5_ *Pn*(NH)_2_ defect antiperovskites. The extrapolation of the linear part is shown for the determination of the optical band gaps. (c) Raman spectra of *AE*_5_ *Pn*_2_(NH)_2_. The region between 3000–3150 cm^−1^ shows the imide stretching vibration (*v*_s_) [49, 50].

To further verify the presence of the imide‐group in *AE*_5_ *Pn*_2_(NH)_2_; Raman spectroscopy measurements were carried out on powder samples (Figure 5c). The region between 3000–3150 cm^−1^ typically shows the stretching vibration *v_s_* (NH^2−^). In the case of Sr_5_Bi_2_(NH)_2_ no successful measurements could be conducted due to the supposed metallic nature of the compound. Nevertheless, the observed *v_s_* (NH^2−^) vibrations of the other *AE*_5_ *Pn*_2_(NH)_2_ compounds show Raman shifts comparable to our previous work [37] and the *AE*NH‐compounds [49, 50]. The observed characteristic wavenumbers assigned to the imide stretching vibrations decrease with increasing *AE* ionic radii from 3088 cm^−1^ (*v_s_* (CaSb/NH^2−^)) to 3046 cm^−1^ (*v_s_* (SrSb/NH^2−^)) [50]. Substituting the *B*‐site with heavier homologues also shifts the *v_s_* (NH^2−^) vibrations to lower wavenumbers from 3102 cm^−1^ (*v_s_* (CaAs/NH^2−^)) over 3088 cm^−1^ (*v_s_* (CaSb/NH^2−^)) to 3076 cm^−1^ (*v_s_* (CaBi/NH^2−^)), as the corresponding force constant of the imide group decreases with increasing ionic radii [50].

### 2.7 Optical Properties

**FIGURE 6:**
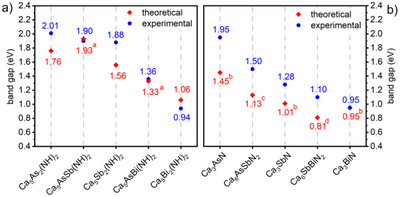
Experimental (UV/Vis) and theoretical band gap energies (HSE+SOC) of (a) Ca_5_ *Pn*_2_(NH)_2_, Ca_5_As*Pn*(NH)_2_ defect antiperovskites and (b) Ca_3_ *Pn*N, Ca_6_ *PnPn*´N_2_ antiperovskites. ^a^Ref.[37]; ^b^Ref.[16]; ^c^Ref.[5]; ^d^Ref.[11].

To determine the optical properties, UV/Vis‐measurements in reflection mode were conducted and quasi absorption spectra were calculated. The band gap values were then determined by extrapolating the absorption edges in the Tauc‐plots with a linear fit as depicted in Figure 5a, b. The UV/Vis measurements of the *AE*_5_ *Pn_2_* (NH)_2_ defect antiperovskite compounds show band gaps of direct nature and exhibit band gap energies ranging from 0.78–2.01 eV, see Figure 6. For Sr_5_Bi_2_(NH)_2_, the band gap energy could not be determined either due to a very low band gap energy or metallic nature of the compound. However, the band gaps obtained from XES/XANES and optical measurements are different based on slightly different probing mechanisms, very different probing depths and experimental conditions. In particular, the attenuation depth and hence the probing volume differ significantly between these techniques [51]. While XES and XANES are element‐specific and sensitive to the partial occupied and unoccupied density of states, optical measurements directly probe the electronic transitions from VB to CB influenced by excitonic effects. Therefore, the values obtained from different techniques can be compared but one should not expect the same values.

In the series of Ca_5_ *Pn_2_* (NH)_2_ compounds, a shift of the band gap values to lower energies can be observed (2.01>1.88>0.94 eV), when the *A*‐site atom is replaced by a heavier homologue (from As^3+^ to Sb^3+^ to Bi^3+^). This coincides with the decreasing network tilt Δ*θ*. Substitution of the *X*‐site cation Ca^2+^ with its heavier homologue Sr^2+^ furthermore shows an enhanced effect in decreasing the band gap energy, with a change of 1.10 eV between Ca_5_Sb_2_(NH)_2_: (*E_g_ *= 1.88 eV) and Sr_5_Sb_2_(NH)_2_ (*E_g_ *= 0.78 eV). Substituting the *B*‐site with N^3−^ instead of NH^2−^ decreases the band gaps in all cases with the biggest decrease of 0.60 eV in Ca_5_Sb_2_(NH)_2_ versus Ca_3_SbN. Additionally, the series of *AE*_3_ *Pn*N compounds shows a similar decrease of the band gap values as seen for the imide compounds, when the *A*‐site is substituted by heavier homologues, which is also in good agreement with previous theoretical calculations in the literature [5, 16]. To showcase the tunability of the band gap energies, we extended the ternary nitride system with the solid solutions Ca_6_AsSbN_2_ (Ca_6_As_2‐x_Sb_x_N_2_, *x* = 0.99(2)) and Ca_6_SbBiN_2_ (Ca_6_Sb_2‐x_Bi_x_N_2_, x = 1.06(4)). These materials show band gap values of 1.50 eV (Ca_6_AsSbN_2_) and 1.10 eV (Ca_6_SbBiN_2_), which are in both cases between the respective ternary compounds. Similarly, the experimental band gaps of the previously reported quaternary defect imide antiperovskites Ca_5_AsSb(NH)_2_ and Ca_5_AsBi(NH)_2_, both lie between the respective ternary imide antiperovskites, highlighting the tunability of the band gaps by gradually changing the pnictide element in both the imide and nitride antiperovskite systems. All measured and calculated band gaps reported in this work are tabulated in Tables S16, S17.

### 2.8 Density of States and Optical Absorption Spectra

**FIGURE 7:**
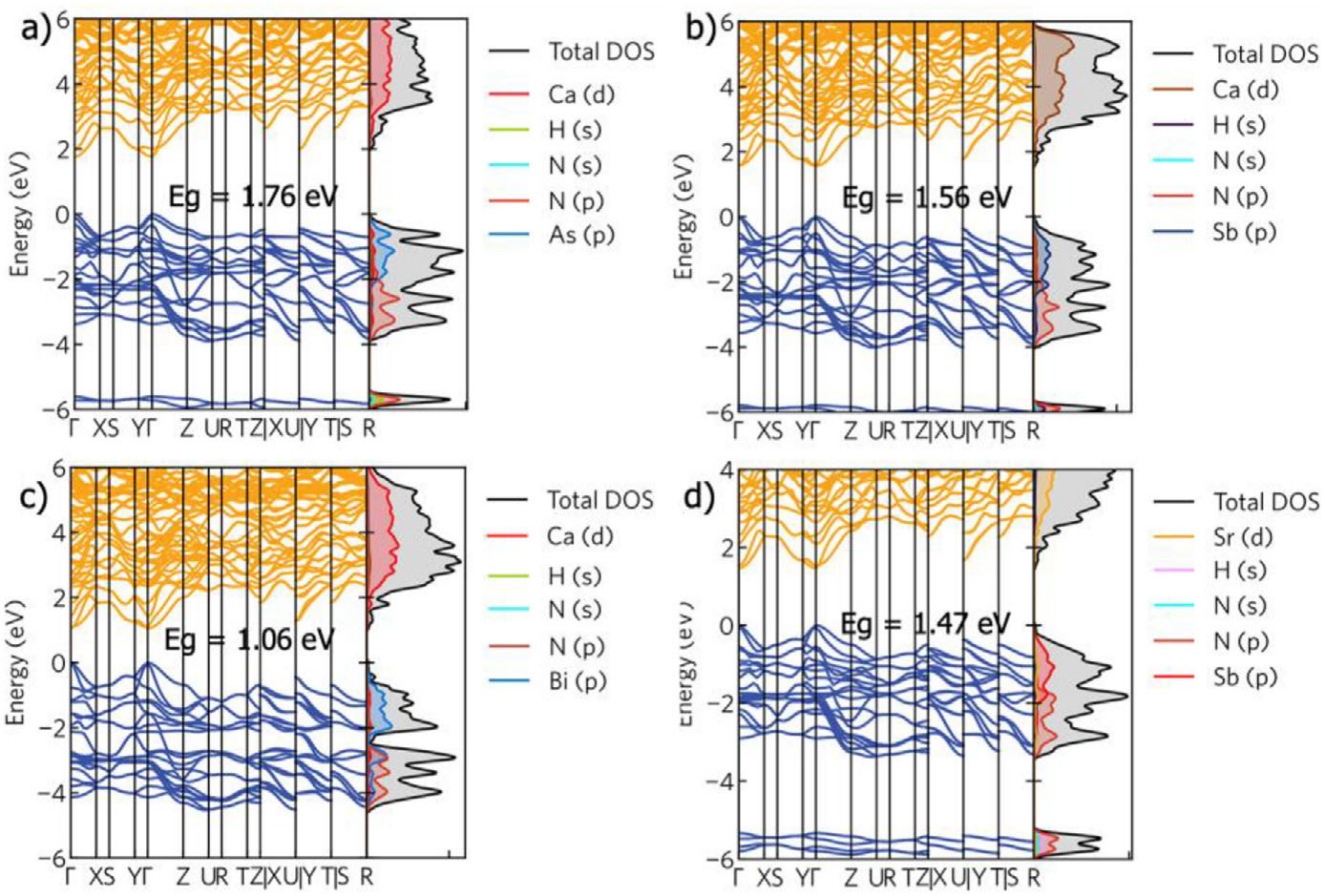
Band structures and density of states of (a) Ca_5_As_2_(NH)_2_, (b) Ca_5_Sb_2_(NH)_2_, (c) Ca_5_Bi_2_(NH)_2_, and (d) Sr_5_Sb_2_(NH)_2_ calculated at the HSE + SOC level.

To evaluate the band gaps obtained from UV/Vis measurements, we employed hybrid functional within the Vienna Ab initio Simulation Package (VASP). Ca_5_As_2_(NH)_2_, Ca_5_Sb_2_(NH)_2_, Sr_5_Sb_2_(NH)_2_, Ca_5_Bi_2_(NH)_2_, and Sr_5_Bi_2_(NH)_2_ exhibit direct band gaps with the valence band maximum (VBM) and conduction band minimum (CBM) located at the Γ point, as shown in Figures 7 and S8. The calculated band gaps of Ca_5_As_2_(NH)_2_, Ca_5_Sb_2_(NH)_2_, Sr_5_Sb_2_(NH)_2_, and Ca_5_Bi_2_(NH)_2_ at the HSE+SOC level are 1.76, 1.56, 1.47, 1.06, and 0.87 eV, respectively, which agree well with the experimentally measured band gaps.

**FIGURE 8:**
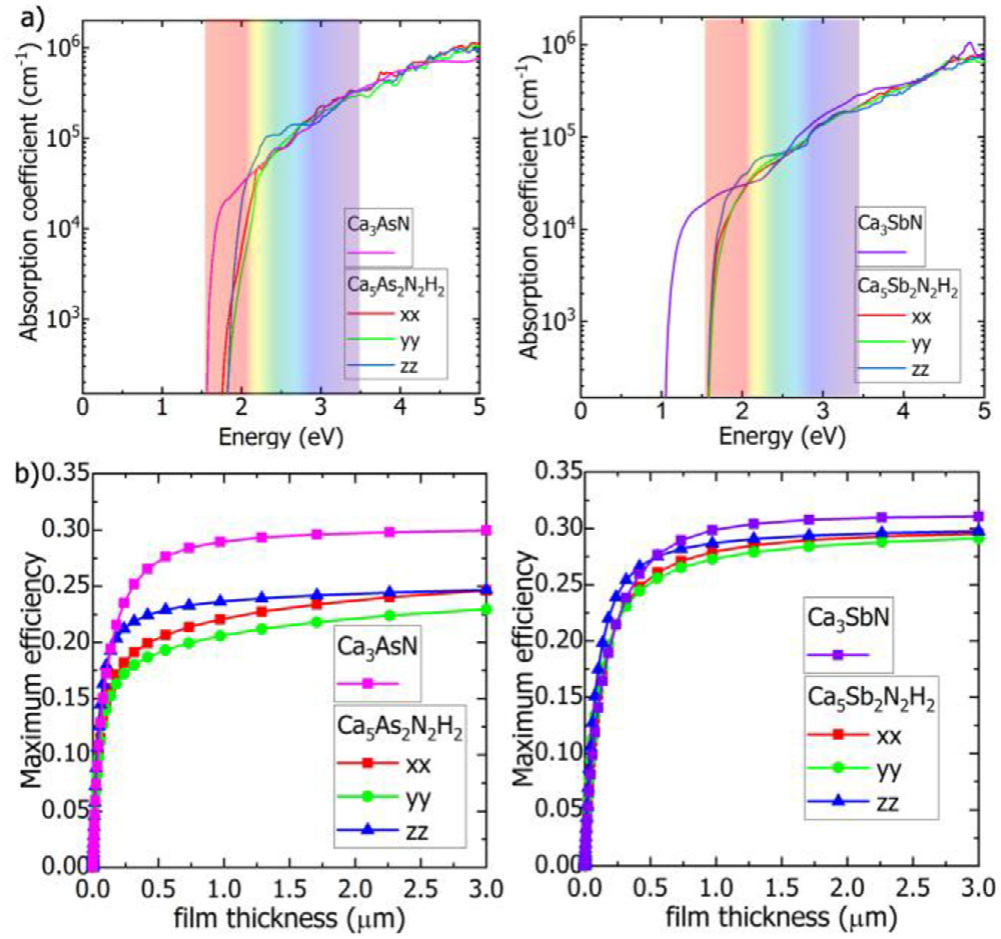
(a) Optical absorption spectra and (b) SLME as a function of film thickness of Ca_5_As_2_(NH)_2_ and Ca_5_Sb_2_(NH)_2_.

When the *X*‐site elements become heavier, moving from Ca to Sr, the band gap decreases. Additionally, when the *A*‐site changes from As to Sb or from Sb to Bi, the band gaps are decreased further. As shown in the projected density of states (Figures 7 and S7), the VBM is predominantly composed of pnictogen (N/As/Sb/Bi) *p*‐orbitals and the CBM is contributed by alkali metals (Ca/Sr) *d*‐orbitals. Thus, varying the *A*‐site and *X*‐site elements would effectively change the band gap size. It is noteworthy that despite the defective octahedral network, all four compounds show dispersive band edges when introducing the imide group. This leads to small effective masses of hole and electron (see Table S18). Ca_5_As_2_(NH)_2_ and Ca_5_Sb_2_(NH)_2_ show high optical absorption coefficients. Both display sharp absorption around the band edges along different directions, and their optical absorption coefficient can reach 10^5^ cm^−1^ in the visible light region, which is comparable to their nitride counterparts, as displayed in Figure 8a.

In addition to the optical absorption spectra, the theoretical conversion efficiency [spectroscopic limited maximum efficiency (SLME)] was investigated. As depicted in Figure 8b, a calculated efficiency exceeding 20% and 25% can be obtained in absorber layers with 0.3 µm thickness for Ca_5_As_2_(NH)_2_ and Ca_5_Sb_2_(NH)_2_, respectively. These result from the relatively rapid optical absorption increase around the band edge. Sr_5_Sb_2_(NH)_2_ and Sr_5_Bi_2_(NH)_2_ show similar optical absorption behavior except a shifting of the absorption onset of Sr_5_Bi_2_(NH)_2_ to the infrared region, as shown in Figure S7a. The calculated efficiency for Sr_5_Sb_2_(NH)_2_ and Sr_5_Bi_2_(NH)_2_ is around 29% and 28%, respectively, with 0.3 µm thin‐film thickness (see Figure S7b).

## 3 Conclusion

In this study, we synthesized a series of new defect antiperovskites‐related imides, namely Ca_5_As_2_(NH)_2_, Ca_5_Sb_2_(NH)_2_, Ca_5_Bi_2_(NH)_2_, Sr_5_Sb_2_(NH)_2_, and Sr_5_Bi_2_(NH)_2_, using the ammonothermal method. The compounds can be regarded as the ternary boundary phases to our recently reported quaternary compounds *AE*_5_As*Pn*(NH)_2_ (*AE* = Ca, Sr; *Pn* = Sb, Bi). The crystal structures were elucidated by scXRD, and pXRD and consist of *AE*_5_NH‐units, with channels of vacancies along the [001] direction. Similar to the antiperovskite *X*_3_ *AB* parental structure an octahedral network tilting is observed following the ionic radii size ratio between the *X* and *A* site ion, despite one missing *X* site. SEM‐EDX and Raman measurements further confirmed the structures. The optical properties of the title compounds were examined using UV/VIS measurements as well as DFT calculations, revealing direct band gaps with energies in the range of 0.78–2.01 eV, suggesting suitability for photovoltaic absorption purposes. XES and XANES measurements at the O and N‐K‐edges probe the band gap and reveal the presence of O in form of the secondary oxide phases *AE*_4_ *Pn*_2_O. High‐temperature pXRD measurements demonstrated the further reaction to the corresponding *AE*_3_ *Pn*N compounds, which were also recently reported to exhibit excellent optoelectronic properties. The presented compounds further broaden the antiperovskite compound space and highlight their structural flexibility.

## Conflicts of Interest

The authors declare no conflicts of interest.

## Supporting information




**Supporting File 1**: The authors have cited additional references within the Supporting Information [52–72].

## Data Availability

The data that supports the findings of this study are available in the supporting information of this article.
