# Supplementary material for: Band Gap Tuneability in Antiperovskite‐Based Nitrides AE 3 PnN and Imides AE 5 Pn 2(NH)2 (AE = Ca, Sr; Pn = As, Sb, Bi)
Source: Angew Chem Int Ed Engl. 2026 Apr 1;65(20):e1423389. doi: 10.1002/anie.1423389 (PMC13159398; doi:10.1002/anie.1423389)
Supplement: Supplementary file 1 — Supporting File 1: The authors have cited additional references within the Supporting Information [52–72]. [file ANIE-65-e1423389-s001.pdf]

Supporting Information

Table of contents

1. **Supplementary Methods**
   - 1.1 Synthesis Details
   - 1.2 Single Crystal X-ray diffraction
   - 1.3 Powder X-ray diffraction
   - 1.4 IR- and Raman Spectroscopy
   - 1.5 UV/VIS Spectroscopy
   - 1.6 SEM/EDX Spectroscopy
   - 1.7 XES and XANES Spectroscopy
   - 1.8 DFT Calculations
   - 1.9 Detailed Single crystal data
   - 1.10 Detailed Rietveld refinement data
   - 1.11 Selected Distances
   - 1.12 EDX-measurements
   - 1.13 Band gaps
2. **Supplementary calculation**
   - 2.1 Oxygen impurities
   - 2.2 Density of states and Electron density map
   - 2.3 Optical absorption spectra and SLME
   - 2.4 Effective mass of hole and electrons
   - 2.5 Band structures and densities of states of $Sr_5Bi_2(NH)_2$

1. Supplementary Methods

1.1 Synthesis Details

Ammonothermal Synthesis

***EA*$_5$*Pn*$_2$(NH)$_2$** compounds were synthesized from Ca_3N_2 (Merck, 99%), CaH_2 (Sigma Aldrich, 99.99%), SrH_2 (Materion, 99.5%), As (Alfa Aeser, 99.9%), Sb (Riedel-de Haen, 99%, 6088.0 mg, 50 mmol) and Bi (Merck, 99%). For the ammonothermal condition NH_3 (5.0, Air liquide) custom made ammonothermal autoclave (autoclave volume: 10 ml, Haynes 282) and Niobium liners were used as reaction vessels. The starting materials were ground in an agate mortar and placed in the autoclave within an open Niobium liner. Ammonia was condensed into the autoclave via condensation by a N_2/Ethanol mixture. The reaction vessel was heated using a three-zone oven for an increased control of the temperature gradient. Subsequently, the autoclave was cooled to room temperature and the residual ammonia was removed.[52] All samples were handled inside a glovebox under argon atmosphere due to the high air-sensitivity of the samples (c(O2) < 0.1 ppm, c(H2O) < 1 ppm, UNIlab, MBRAUN, Garching, Germany).

$Ca_5As_2(NH)_2$ was synthesized from CaH_2 (8 mmol, 336.8 mg) and As (2 mmol, 149,8 mg). The autoclave was heated to 400 °C within 2 h and kept at this temperature for 6 min before the temperature was raised to 750°C within 4 h and held for 30 h. An autogenous pressure of 116 MPa was reached after the second heating step. Additionally, KN_3 (1.0 mmol, 81.1 mg) was added as mineralizer to further promote the synthesis of large single crystals. $Ca_5As_2(NH)_2$ was obtained as a brownish crystalline phase next to colorless crystals of $Ca(NH)_2$ and dark red crystals of Ca_4As_2O as a byproduct.

$Ca_5Sb_2(NH)_2$ was synthesized from Ca_3N_2 (1.75 mmol, 259.4 mg) and Sb (2 mmol, 243.5 mg). The autoclave was heated to 400 °C within 2 h and kept at this temperature for 2 h before the temperature was raised to 600°C within 2 h and held for 10 h and raised again to 750°C within 2 h and held for 30 h. An autogenous pressure of 103 MPa was reached after the third heating step. $Ca_5Sb_2(NH)_2$ was obtained as black crystals which appear purple under light. Slight amount of byproducts of CaO and Ca_4Sb_2O were observed as white and red crystals.

$Ca_5Bi_2(NH)_2$ was synthesized using CaH_2 (4.5 mmol, 189.5 mg) and Bi (1.5 mmol, 313.5 mg). The autoclave was heated to 400 °C within 2 h and kept at this temperature for 2 h before the temperature was raised to 600°C within 2 h and held for 12 h and raised again to 750°C within 4 h and held for 60 h. An autogenous pressure of 100 MPa was reached after the third heating step. $Ca_5Bi_2(NH)_2$ was obtained as grey crystals next to metallic crystals of Bi. Slight amounts of Bi made an UVVis measurement not possible. The samples were therefore heated under high vacuum (10^{-5}-10^{-6} Bar) at 560 °C under which the Bi reacted to Ca_3BiN. Further heat treatment using a temperature of >560 °C led to the formation of the intermetallic phases Ca_5Bi_3 and/or $Ca_{11}Bi_{10}$.

$Sr_5Sb_2(NH)_2$ was synthesized from SrH_2 (5 mmol, 448.1 mg) and Sb (2.0 mmol, 243.5 mg). The autoclave was heated to 400 °C within 2 h and kept at this temperature for 13 h before the temperature was raised to 800 °C within 4 h and held for 120 h. An autogenous maximum pressure of 116 MPa was reached after the second heating step. $Sr_5Sb_2(NH)_2$ was obtained as black crystals with Sr_4Sb_2O as a byproduct.

$Sr_5Bi_2(NH)_2$ was synthesized from SrH_2 (3.75 mmol, 318.1 mg) and Bi (1 mmol, 209.0 mg). The autoclave was heated to 400 °C within 2 h and kept at this temperature for 12 h before the temperature was raised to 800 °C within 4 h and held for 20 h. An autogenous maximum pressure of 53 MPa was reached after the second heating step. $Sr_5Bi_2(NH)_2$ was obtained as grey crystals with colorless SrO and SrNH as byproducts.

1.2 Single crystal X-ray Diffraction

Single crystal X-ray data of $AE_5Pn_2(NH)_2$ (*AE* = Ca, Sr; *Pn* = As, Sb, Bi) were collected using a Bruker D8 Venture. All Crystals were prepared in glass capillaries (Hilgenberg GmbH, Germany) under dried paraffin oil. For the data collection, indexing, data reduction and absorption correction (multi scan) APEX 3 software[53] was used. The XPREP[54] software was used for the analysis of the systematically absent reflections and space group determination. Structure solutions were carried out in WINGX[55] using SHELXT.[56] For the refinement the SHELXLE software[57] was used. All atoms were refined anisotropically with the exception of the H-atoms, which were refined isotropical.
Single crystal X-ray diffraction data of $Ca_5As_2(NH)_2$, $Ca_5Sb_2(NH)_2$, $Ca_5Bi_2(NH)_2$, $Sr_5Sb_2(NH)_2$ and $Sr_5Bi_2(NH)_2$ were deposited under CSD number 2517683-2517687.[39] These data are provided free of charge by the joint Cambridge Crystallographic Data Centre and Fachinformationszentrum Karlsruhe Access Structures service www.ccdc.cam.ac.uk/structures.

1.3 Powder X-ray Diffraction

Powder X-ray diffraction data were collected using a STOE StadiP diffractometer equipped with Cu-$K_{\alpha1}$ (λ = 1.5406 Å) radiation, a Ge(111) monochromator and a DECTRIS MYTHEN 1K Si-strip detector in modified Debye-Scherrer geometry. Samples were sealed in glass capillaries (Hilgenberg GmbH, Germany). Due to the high absorption of samples containing Sr and/or Bi, amorphous Boron (Sigma Aldrich, ≥95%) was added for the preparation of the capillaries. Topas6[58] was used with a fundamental parameters refinement approach for the Rietveld refinement of the obtained data. All atoms were refined isotropic and the background was described using the Shifted Chebyshev polynomial with 10 parameters.

1.4 Raman Spectroscopy

Raman measurements were conducted on a Bruker Vertex 70 spectrometer with a Ram II FTIR/Raman extension equipped with a Coherent 1064 nm laser with a maximum power of 500 mW and a liquid nitrogen cooled LN-Ge NIR-detector. Spectra were recorded in reflectance with the laser defocused and with different laser intensities between 10 mW and 100 mW to prevent thermal degradation of the sample. Air-sensitive samples were encapsulated inside a N_2-filled glove box between two transparent glass plates using a solvent-free two-component adhesive (Torr Seal Epoxy Resin, Agilent Technologies, Inc., USA). Spectra were baseline corrected, using the “Concave rubberband correction” method.

1.5 UV/Vis Spectroscopy

UV/Vis measurements were performed on a Perkin Elmer Lambda 1050 UV/VIS/NIR spectrophotometer equipped with a 150 mm integrating sphere and a custom-built spot reduction kit. Air-sensitive samples were encapsulated inside a N_2-filled glove box between two transparent glass plates using a solvent-free two-component adhesive (Torr Seal Epoxy Resin, Agilent Technologies, Inc., USA). Spectra were recorded in reflectance. TAUC-Plots were calculated using the Kubelka-Munk function.[59]

1.6 SEM/EDX Spectroscopy

Direct SEM image preparation was done using an FEI Helios Nanolab G3 UC, equipped with a Schottky-type field-emitter, operated between 1 and 30 kV. All measurements showed a high degree of oxygen content, which should not be taken into account as the sample is exposed to air for the measurement preparation.

1.7 XES and XANES Spectroscopy

The bulk electronic properties of $AE_5Pn_2(NH)_2$ were investigated using a combination of soft X-ray emission spectroscopy (XES) and X-ray absorption near-edge spectroscopy (XANES). XES and XANES are element-specific techniques that probe the partial occupied and unoccupied density of states (PDOS), respectively, governed by the dipole selection rule ($\Delta l = \pm 1$). These measurements were carried out at the REIXS beamline at the Canadian Light Source.[60] The partial fluorescence yield (PFY) absorption spectra were recorded by promoting core electrons into the conduction band and detecting their subsequent decays using an energy-dispersive silicon drift detector (SDD). Emission spectra were measured by exciting core electrons non-resonantly into continuum states using incident photon energies well above the binding energy of the target element, and detecting the emitted X-rays using a Rowland-type grating spectrometer, providing an effective energy resolution of 450 meV. XANES measurements with energy resolutions (ΔE) of 0.125 eV and 0.08 eV for the oxygen and nitrogen K-edges, respectively, were normalized during acquisition using the photocurrent from a gold mesh positioned upstream of the sample to monitor variations in the incident photon flux. All measurements were performed at room temperature, with the incident X-ray beam directed at an angle of 62.5° relative to the sample surface.

For all soft X-ray measurements, powder samples were pressed on the freshly scraped indium foil (to eliminate surface oxides) mounted on a stainless steel sample plate. Sample preparation was carried out inside a glove bag before being transferred into an ultrahigh vacuum (1.0×10^{-9} Torr) measurement chamber. The XANES spectra were calibrated using the first peak of reference compounds: hexagonal boron nitride (h-BN) at 402.1 eV for the nitrogen K-edge, and bismuth germanium oxide (BGO) at 532.7 eV for the oxygen K-edge. The XES spectra were calibrated using the elastic scattering features of the incident beam to ensure alignment with the XANES data on a common energy scale.

The XANES spectra are influenced by the presence of a core hole, which typically shifts the conduction band edge to slightly lower energies. Consequently, a direct determination of the band gap from the XES and XANES onsets requires correction by adding a core-hole shift. The core-hole shift was determined by calculating the energy difference between the conduction band onset in ground-state and core-hole-state calculations. The resulting band gaps are listed in Table S16.

1.8 DFT Calculations

The first-principles calculations were performed using the Vienna Ab initio Simulation Package (VASP).[61] The generalized gradient approximation (GGA) in the Perdew-Burke-Ernzerhof (PBE) form was used for the exchange-correlation functional.[62] The convergence criteria for the energy and force were set to 10^{-4} eV and 0.02 eV/Å, respectively. The kinetic-energy cutoff was chosen as 520 eV. For the Brillouin zone integration, the Monkhorst−Pack k-point mesh with a grid spacing of $\sim 2\pi \times 0.03$ Å^{-1} was used. For optical absorption spectra, a high-density k-point mesh (less than $2\pi \times 0.02$ Å^{-1}) was employed for the calculation. Hybrid functional (HSE) with the 25% nonlocal Fock exchange was employed for the band structure calculations.[63] The SOC effect was taken into account as it affects strongly the electronic structure of compounds containing a heavy p-electron element like Sb, Bi.[64-65] Postprocessing of electronic structures was carried out by sumo.[66] The theoretical conversion efficiency of the solar cells as a function of thickness of the absorber layer was calculated based on the method proposed by Yu, L. et al. and a python code (SL3ME).[67] The calculated band gaps from VASP were obtained using the experimental lattice and structural parameters after geometry relaxation by DFT.

The electronic properties of the N and O K-edges of $AE_5Pn_2(NH)_2$ (*AE* = Ca, Sr; *Pn* = Bi, Sb) were calculated using the WIEN2K software package, which employs the full potential linearized augmented plane wave method with local orbitals (LAPW + lo) to self-consistently solve the Kohn-Sham equations.[68] For the exchange-correlation potential, the Perdew−Burke−Ernzerhof (PBE) variant of the generalized gradient approximation (GGA) was used.[62] However, since this functional is well known to underestimate the band gap by up to 50% due to fundamental limitations of standard DFT,[69-70] the modified Becke-Johnson (mBJ) exchange-correlation functional was employed to obtain a more accurate estimation of the band gaps. DFT calculations were used to compute the density of states (DOS), XES and XANES spectra, and the electronic band gap. The calculated XES and XANES spectra were obtained by multiplying the PDOS with the dipole and radial transition matrix elements.[71] In the final state of the measured K-edge XANES spectra, a core-hole is present in the 1s state, and its presence distorts the ground states. To account for the presence of a 1s core-hole in the final state of the measured K-edge XANES spectra, a 2 × 1 × 3 supercell was created, in which one core electron (1s) was removed from the target atom and a compensating background lattice charge was added to the supercell to maintain charge neutrality. For unit cell calculations, 8 × 4 × 12 k-point mesh was used along with $R_{MT} K_{max}$ of 3.0 and energy separation of -6.0 Ry between core and valence states. These parameters yielded a total energy convergence of 10^{-5} Ry and a charge convergence of 10^{-4} e. In contrast, for the 2 × 1 × 3 supercell calculations, the k-point mesh was reduced to 4 × 4 × 4. To facilitate comparison with experimental spectra, the calculated spectra were broadened using a combination of Lorentzian functions to account for core-hole lifetime broadening and Gaussian functions to account for instrumental broadening.[72]

1.9 Detailed Single Crystal data

Table S1: Detailed Single crystal-data of $Ca_5As_2(NH)_2$, $Ca_5Sb_2(NH)_2$, $Ca_5Bi_2(NH)_2$, $Sr_5Sb_2(NH)_2$ and $Sr_5Bi_2(NH)_2$.

formula	$Ca_5As_2(NH)_2$	$Ca_5Sb_2(NH)_2$	$Ca_5Bi_2(NH)_2$	$Sr_5Sb_2(NH)_2$	$Sr_5Bi_2(NH)_2$
crystal system			orthorhombic		
space group			*Pbam* (No.55)		
formula weight / g*mol^{-1}	380.28	473.94	648.40	711.64	886.10
a / Å	6.6586(11)	6.792(2)	6.8616(2)	7.2261(6)	7.2864(14)
b / Å	12.654(2)	13.315(4)	13.4732(5)	13.9816(12)	14.138(3)
c / Å	4.8211(9)	4.9177(12)	4.9449(2)	5.2195(4)	5.2458(9)
V / Å^3	406.22(12)	444.7(2)	457.14(3)	527.42(7)	540.4(2)
Z	2	2	2	2	2
Diffractometer			Bruker D8 Venture		
Radiation type			$Mo_{K\alpha}$ (0.71074 nm)		
Temperature / K			293		
Goodness of fit (Goof)	1.354	1.007	1.162	1.058	1.146
µ / mm^{-1}	11.290	8.874	41.164	30.064	56.862
Calculated X-ray density / g·cm^{-3}	3.109	3.539	4.710	4.481	5.446
F(000)	364	436	564	616	744
ϑ range / °	3.220 < 2ϑ <27.970	3.060 < 2ϑ <24.979	3.024 < 2ϑ <39.380	2.913 < 2ϑ <30.503	3.145 < 2ϑ <33.1240
Total no. of reflections	4459	11332	16792	10681	16433
Independent reflections [I≥2σ(I) / all]	397	438	1204	889	1122
R_1 ($F^2 \geq 2\sigma(F^2)$ / all)	0.0257 / 0.0326	0.0079 / 0.0084	0.0148/ 0.0188	0.0196/ 0.0226	0.0209/ 0.0253
wR_2 ($F^2 \geq 2\sigma(F^2)$ / all)	0.0568 / 0.0627	0.0153 / 0.0153	0.0240 / 0.0246	0.0419 / 0.0428	0.0353 / 0.0359
R_{int} / R_σ	0.0547 / 0.0291	0.0318 / 0.0153	0.0428/ 0.0189	0.0461/ 0.0243	0.0624/ 0.0281
$\Delta\rho_{max}$; $\Delta\rho_{min}$ / e*Å^{-3}	0.951; –1.146	0.338; –0.343	1.317; –1.357	0.985; –0.899	1.424; –1.549

Table S2: Atomic coordinates, Occupancy, Wyckoff symbol and equivalent displacement parameters (Å^2) of $Ca_5As_2(NH)_2$ from single crystal data.

Atom	Wyckoff.	*x*	*y*	*z*	U_{eq}	s.o.f
As1	4*h*	0.30857(10)	0.36567(5)	1/2	0.0089(3)	1
Ca2	4*g*	0.0908(2)	0.27023(10)	0	0.0109(3)	1
Ca3	2*a*	0	0	0	0.0120(4)	1
Ca4	4*h*	0.2998(2)	0.10968(10)	1/2	0.0134(4)	1
N5	4*g*	0.2894(8)	0.1214(4)	0	0.0062(11)	1
H6	4*g*	0.351(9)	0.058(3)	0	0.01(2)	1

Table S3: Anisotropic displacement parameters (Å^2) of $Ca_5As_2(NH)_2$ from single-crystal data.

Atom	U_{11}	U_{22}	U_{33}	U_{12}	U_{13}	U_{23}
As1	0.0099(4)	0.0084(4)	0.0084(4)	-0.0014(3)	0.00000	0.00000
Ca2	0.0067(7)	0.0113(7)	0.0148(7)	0.0038(5)	0.00000	0.00000
Ca3	0.0117(9)	0.0086(10)	0.0158(10)	-0.0041(7)	0.00000	0.00000
Ca4	0.0206(8)	0.0146(7)	0.0051(7)	0.0001(6)	0.00000	0.00000
N5	0.009(3)	0.002(3)	0.008(3)	-0.002(2)	0.00000	0.00000

Table S4: Atomic coordinates, Occupancy, Wyckoff symbol and equivalent displacement parameters (Å^2) of $Ca_5Sb(NH)_2$ from single crystal data.

Atom	Wyckoff.	*x*	*y*	*z*	U_{eq}	s.o.f
Sb1	4*h*	0.29428(2)	0.36703(2)	1/2	0.00807(6)	1
Ca1	2*a*	0	0	0	0.01021(12)	1
Ca2	4*g*	0.06080(5)	0.25960(3)	0	0.00946(9)	1
Ca3	4*h*	0.29692(5)	0.11108(3)	1/2	0.01022(9)	1
N1	4*g*	0.2749(2)	0.12429(11)	0	0.0076(3)	1
H1	4*g*	0.350(4)	0.077(2)	0	0.021(6)	1

Table S5: Anisotropic displacement parameters (Å^2) of $Ca_5Sb(NH)_2$ from single-crystal data.

Atom	U_{11}	U_{22}	U_{33}	U_{12}	U_{13}	U_{23}
Sb1	0.00824(8)	0.00794(8)	0.00804(8)	-0.00082(5)	0.00000	0.00000
Ca1	0.0115(3)	0.0093(3)	0.0098(3)	-0.0034(2)	0.00000	0.00000
Ca2	0.0077(2)	0.0098(2)	0.0109(2)	0.00221(14)	0.00000	0.00000
Ca3	0.0121(2)	0.0117(2)	0.0068(2)	-0.0012(2)	0.00000	0.00000
N1	0.0078(8)	0.0061(8)	0.0091(8)	0.0013(6)	0.00000	0.00000

Table S6: Atomic coordinates, Occupancy, Wyckoff symbol and equivalent displacement parameters (Å^2) of $Ca_5Bi_2(NH)_2$ from single crystal data.

Atom	Wyckoff.	*x*	*y*	*z*	U_{eq}	s.o.f
Bi1	4*g*	0.28990(2)	0.36807(2)	0	0.00945(3)	1
Ca1	4*g*	0.20618(9)	0.61207(4)	0	0.01141(9)	1
Ca2	4*h*	0.05523(8)	0.25842(4)	1/2	0.01053(9)	1
Ca3	2*b*	1/2	1/2	1/2	0.01126(14)	1
N01	4*h*	0.2707(3)	0.12587(16)	1/2	0.0087(3)	1
H1	4*h*	0.356(8)	0.073(4)	1/2	0.04(2)	1

Table S7: Anisotropic displacement parameters (Å^2) of $Ca_5Bi_2(NH)_2$ from single-crystal data.

Atom	U_{11}	U_{22}	U_{33}	U_{12}	U_{13}	U_{23}
Bi1	0.00947(4)	0.00932(4)	0.00955(4)	-0.00084(4)	0.00000	0.00000
Ca1	0.0133(2)	0.0129(2)	0.0081(2)	0.0018(2)	0.00000	0.00000
Ca2	0.0080(2)	0.0113(2)	0.0124(2)	0.0021(2)	0.00000	0.00000
Ca3	0.0118(3)	0.0109(3)	0.0111(3)	0.0032(3)	0.00000	0.00000
N01	0.0091(9)	0.0088(8)	0.0082(8)	0.0018(8)	0.00000	0.00000

Table S8: Atomic coordinates, Occupancy, Wyckoff symbol and equivalent displacement parameters (Å^2) of $Sr_5Sb(NH)_2$ from single crystal data.

Atom	Wyckoff.	*x*	*y*	*z*	U_{eq}	s.o.f
Sb01	4*h*	0.30407(4)	0.36546(2)	1/2	0.01268(9)	1
Sr1	2*a*	0	0	0	0.01477(13)	1
Sr2	4*g*	0.07252(5)	0.26383(3)	0	0.01404(10)	1
Sr3	4*h*	0.30157(6)	0.10888(3)	1/2	0.01545(10)	1
N	4*g*	0.2788(5)	0.1244(3)	0	0.0130(7)	1
H	4*g*	0.323(9)	0.059(5)	0	0.05(2)	1

Table S9: Anisotropic displacement parameters (Å^2) of $Sr_5Sb(NH)_2$ from single-crystal data.

Atom	U_{11}	U_{22}	U_{33}	U_{12}	U_{13}	U_{23}
Sb01	0.01238(13)	0.01205(14)	0.0136(2)	-0.00143(9)	0.00000	0.00000
Sr1	0.0155(3)	0.0130(3)	0.0158(3)	-0.0041(2)	0.00000	0.00000
Sr2	0.01034(17)	0.0144(2)	0.0174(2)	0.00235(14)	0.00000	0.00000
Sr3	0.0178(2)	0.0163(2)	0.0123(2)	-0.0017(2)	0.00000	0.00000
N	0.014(2)	0.013(2)	0.012(2)	0.0011(12)	0.00000	0.00000

Table S10: Atomic coordinates, Occupancy, Wyckoff symbol and equivalent displacement parameters (Å^2) of $Sr_5Bi_2(NH)_2$ from single crystal data.

Atom	Wyckoff.	*x*	*y*	*z*	U_{eq}	s.o.f
Bi1	4*h*	0.30023(3)	0.36618(2)	1/2	0.01166(6)	1
Sr1	2*a*	0	0	0	0.01301(13)	1
Sr2	4*g*	0.06701(6)	0.26245(4)	0	0.01272(10)	1
Sr3	4*h*	0.29986(7)	0.10948(3)	1/2	0.01413(9)	1
N01	4*g*	0.2739(6)	0.1258(3)	0	0.0109(7)	1
H01	4*g*	0.326(16)	0.081(9)	0	0.10(5)	1

Table S11: Anisotropic displacement parameters (Å^2) of $Sr_5Bi_2(NH)_2$ from single-crystal data.

Atom	U_{11}	U_{22}	U_{33}	U_{12}	U_{13}	U_{23}
Bi1	0.01225(8)	0.01148(9)	0.01126(9)	-0.00132(6)	0.00000	0.00000
Sr1	0.0141(3)	0.0124(3)	0.0125(3)	-0.0039(2)	0.00000	0.00000
Sr2	0.0094(2)	0.0138(2)	0.0150(2)	0.0023(2)	0.00000	0.00000
Sr3	0.0164(2)	0.0159(2)	0.0101(2)	-0.0023(2)	0.00000	0.00000
N01	0.010(2)	0.010(2)	0.013(2)	0.0013(14)	0.00000	0.00000

1.10 Detailed Rietveld refinement data

Table S12: Detailed Rietveld refinement data of $AE_5Pn_2(NH)_2$: $Ca_5As_2(NH)_2$, $Ca_5Sb_2(NH)_2$, $Ca_5Bi_2(NH)_2$, $Sr_5Sb_2(NH)_2$ and $Sr_5Bi_2(NH)_2$.

formula	$Ca_5As_2(NH)_2$	$Ca_5Sb_2(NH)_2$	$Ca_5Bi_2(NH)_2$	$Sr_5Sb_2(NH)_2$	$Sr_5Bi_2(NH)_2$
crystal system			orthorhombic		
space group			*Pbam* (no.55)		
formula weight / g*mol^{-1}	380.28	473.94	648.40	711.64	886.10
a / Å	6.64854(5)	6.8080(3)	6.86425(7)	7.23256(8)	7.29190(7)
b / Å	12.6515(1)	13.3250(5)	13.4749(2)	14.0047(2)	14.1654(2)
c / Å	4.81572(5)	4.9170(3)	4.94712(6)	5.22186(7)	5.25506(5)
V / Å^3	405.068(6)	446.06(3)	457.582(9)	528.92(1)	542.809(10)
Z			2		
Diffractometer			Stoe STADI P		
Radiation type			Cu-$K_{\alpha 1}$ (1.5406 Å)		
Monochromator			Ge(111)		
Detector			Mythen 1K		
Background function			shifted Chebyshev, 10 polynomials		
Calculated X-ray density / g·cm^{-3}	3.11770(5)	2.77(3)	4.70587(9)	4.4558(10)	5.34(3)
ϑ range / °	$5 < 2\vartheta < 92.195$	$5 < 2\vartheta < 100.06$	$5 < 2\vartheta < 101.68$	$5 < 2\vartheta < 96.755$	$5 < 2\vartheta < 100.81$
Data points	5814	6338	6446	6118	6521
Total no. of reflections	207	267	283	294	332
Refined parameters	49	76	36	62	42
Goodness of fit (Goof)	1.5322	1.029	2.628	1.158	1.121
R_p	0.0643	0.0518	0.0663	0.0584	0.0543
R_{wp}	0.0931	0.0690	0.0951	0.0772	0.0729
R_{Bragg}	0.0522	0.0219	0.0249	0.0290	0.0250

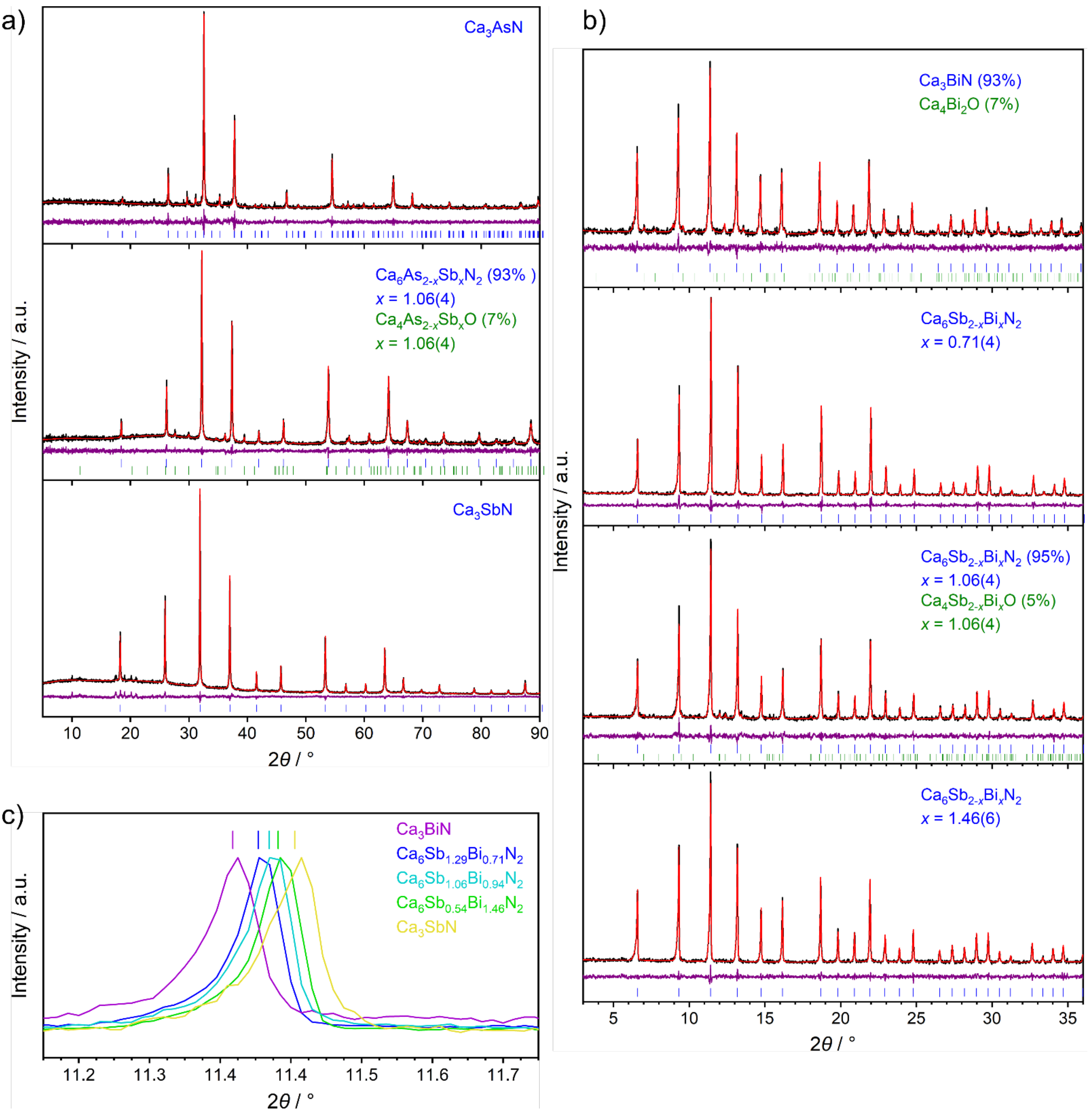

Figure S1. Rietveld refinement of pXRD-data of **a)** Ca_3AsN, $Ca_6As_{2-x}Sb_xN_2$, Ca_3SbN (measured using Cu-$K_{\alpha1}$ (1.5406 Å)) and **b)** of $Ca_6Sb_{2-x}Bi_xN_2$ and Ca_3BiN (measured using Ag-$K_{\alpha1}$ (0.5595 Å)). Black measured data points, red rietveld fit, purple difference plot and colored bars correspond to the phase. Top three pXRD-data were, bottom two pXRD-data with. **c)** shows the [111] reflex of all samples in the Ca_3SbN – Ca_3BiN system, with the refined 2θ-position marked using colored bars.

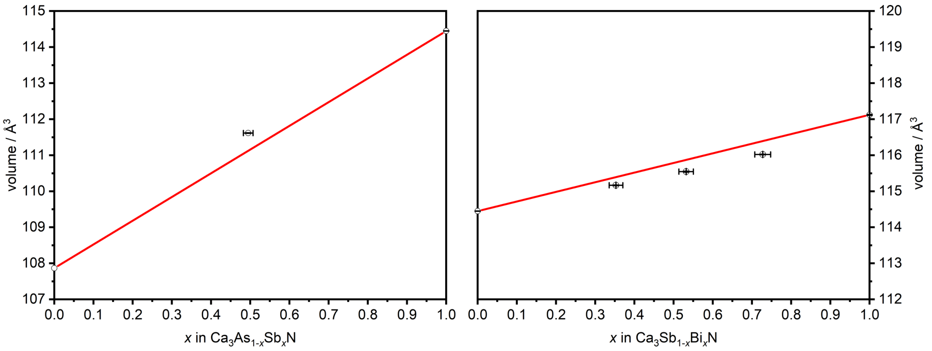

Figure S2. Cell volumes of $Ca_3As_{1-x}Sb_xN$ (x = 0.49(1)) (left) and $Ca_3Sb_{1-x}Bi_xN$ (x = 0.35(2), 0.53(2), 0.73(2)) (right) and their respective edge phases. For Ca_3AsN was the volume determined using the pseudocubic lattice parameters a_{cubic}, b_{cubic} and c_{cubic} with $a_{cubic} = a/\sqrt{2}$, $b_{cubic} = b/\sqrt{2}$ and $c_{cubic} = c/2$ as Ca_3AsN crystallizes in a orthorhombic space group *Pbnm*. Error bars represent the standart deviations obtained by rietveld refinement and the red lines indicate vegard´s law.

Table S13: Detailed Rietveld refinement data of AE_3PnN and $AE_6PnPn'N_2$: Ca_3AsN, Ca_6AsSbN_2, Ca_3SbN, Ca_6AsBiN_2 and Ca_3BiN.

formula	Ca_3AsN	$Ca_6As_{2-x}Sb_xN_2$ X = 0.99(2)	Ca_3SbN	$Ca_6Sb_{2-x}Bi_xN_2$ X = 0.71(4)	X = 1.06(4)	X = 1.46(4)	Ca_3BiN
crystal system	orthorhombic	cubic					
space group	*Pbnm* (no.62)	$Pm\bar{3}m$ (no. 221)					
a / Å	6.727(1)	4.8148(1)	4.8552(6)	4.8653(1)	4.8706(2)	4.8773(1)	4.8927(2)
b / Å	6.725(1)	-	-	-	-	-	-
c / Å	9.5375(5)	-	-	-	-	-	-
V / Å^3	431.50(13)	111.62(1)	114.45(4)	115.17(1)	115.55(1)	116.02(1)	117.12(1)
Z	4	1	1	1	1	1	1
Diffractometer	Stoe STADI P						
Radiation type	Cu-K$_{\alpha1}$ (1.5406 Å)	Ag-K$_{\alpha1}$ (0.5595 Å)					
Monochromator	Ge(111)						
Detector	Mythen 1K						
Background function	shifted Chebyshev, 16 polynomials						
Calculated X-ray density / g·cm^{-3}	3.219(1)	3.46(2)	3.7143(1)	4.13(5)	4.35(5)	4.57(6)	4.866(6)
ϑ range / °	5 < 2ϑ < 92.420	5 < 2ϑ < 100.385	5 < 2ϑ < 90.845	2 < 2ϑ < 37.235	2 < 2ϑ < 36.170	2 < 2ϑ < 37.235	2 < 2ϑ < 36.950
Data points	5829	6360	5724	2350	2279	2350	2331
Total no. of reflections	203	23	21	33	32	33	33
Refined parameters	33	26	24	25	32	24	30
Goodness of fit (Goof)	0.939	0.639	0.861	0.988	0.893	0.886	0.874
R_p	0.1446	0.0693	0.0426	0.0831	0.0966	0.0694	0.0708
R_{wp}	0.1928	0.0929	0.0627	0.1063	0.1241	0.0895	0.0901
R_{Bragg}	0.0267	0.0144	0.0194	0.0210	0.0246	0.0113	0.0142

1.11 Selected Distances

Table S14: Selected distances in $Ca_5As_2(NH)_2$, $Ca_5Sb_2(NH)_2$, $Ca_5Bi_2(NH)_2$, $Sr_5Sb_2(NH)_2$ and $Sr_5Bi_2(NH)_2$ in Å (eq. = equatorial; ap. = apical).

formula	$Ca_5As_2(NH)_2$	$Ca_5Sb_2(NH)_2$	$Ca_5Bi_2(NH)_2$	$Sr_5Sb_2(NH)_2$	$Sr_5Bi_2(NH)_2$	
N(1)-*AE*(1)	2.301(5)	2.315(2)	2.319(2)	2.455(4)	2.451(4)	1x
N(1)-*AE*(2)	2.4161(6)	2.4697(6)	2.4845(2)	2.6240(5)	2.6398(7)	2x
N(1)-*AE*(2)	2.431(5)	2.482(2)	2.499(2)	2.636(4)	2.657(4)	1x
N(1)-*AE*(3)	2.464(5)	2.495(2)	2.515(2)	2.662(4)	2.673(4)	1x
Pn(1)-*AE*(2)	3.0614(10)	3.2568(6)	3.2997(4)	3.4103(4)	3.4523(5)	2x
Pn(1)-*AE*(3)	3.171(2)	3.3080(11)	3.3373(6)	3.4883(6)	3.5162(9)	1x
Pn(1)-*AE*(1)	3.2132(6)	3.3366(6)	3.3691(2)	3.5150(3)	3.5465(4)	2x
Pn(1)-*AE*(3)	3.240(2)	3.3904(10)	3.4144(6)	3.5878(6)	3.6292(9)	1x
Pn(1)-*AE*(3)	3.286(2)	3.4080(11)	3.4492(6)	3.6128(6)	3.6567(9)	1x
Pn(1)-*AE*(3)	3.402(2)	3.4261(10)	3.4680(6)	3.6488(6)	3.6621(9)	1x
Pn(1)-*AE*(2)	3.5071(11)	3.4879(6)	3.5117(4)	3.7206(4)	3.7370(6)	2x
N(1)-H(1)	0.90(4)	0.81(3)	0.92(5)	0.97(7)	0.74(12)	1x
$\bar{d}(N\text{-}AE)_{eq}$	2.4318	2.4791	2.4957	2.6364	2.6524	
$d(N\text{-}AE)_{ap}$	2.3012	2.3152	2.3185	2.4542	2.4505	
$\bar{d}(N\text{-}AE)$	2.4057	2.4463	2.4602	2.5999	2.6120	
$\bar{d}(Pn\text{-}AE)$	3.2662	3.3695	3.4030	3.5630	3.5936	

1.12 EDX-measurements

Table S15: Averaged EDX measurements.

formula	Ca (%)	Sr (%)	As (%)	Sb (%)	Bi (%)	Molar ratio *EA*: *Pn*
$AE_5Pn_2(NH)_2$ (calc)	71.4			28.6		5: 2
$Ca_5As_2(NH)_2$	75(3)	-	25(4)	-	-	5.89: 2
$Ca_5Sb_2(NH)_2$	71(7)	-	-	29(5)-	-	4.83: 2
$Ca_5Bi_2(NH)_2$	71(11)	-	-	-	29(6)	4.87: 2
$Sr_5Sb_2NH)_2$	-	71(4)	-	29(3)	-	4.93: 2
$Sr_5Bi_2(NH)_2$	-	69(2)	-	-	31(2)	4.51: 2

1.13 Band gaps

Table S16: experimental and theoretical band gaps of *AE*$_5$*Pn*$_2$(NH)$_2$ antiperovskite related imides.

formula	$Ca_5As_2(NH)_2$	$Ca_5Sb_2(NH)_2$	$Ca_5Bi_2(NH)_2$	$Sr_5Sb_2(NH)_2$	$Sr_5Bi_2(NH)_2$
E_g (exp_UV/Vis) /eV	2.01	1.88	0.94	0.78	-
E_g (VASP_HSE+SOC) /eV	1.76	1.56	1.06	1.47	0.87
E_g (exp_XES/XANES) /eV	-	2.08 ± 0.42	2.21 ± 0.40	1.55 ± 0.45	-
E_g (Wien2k_PBE) /eV	-	1.16	1.06	1.13	-
E_g (Wien2k_mBJ) /eV	-	1.67	1.57	1.68	-

Table S17: experimental and theoretical band gaps of *AE*$_3$*Pn*N antiperovskites.

formula	Ca_3AsN	Ca_3SbN	Ca_3BiN	Sr_3SbN	Sr_3BiN
E_g (exp_UV/Vis) /eV	1.95	1.28	0.95	1.22	-
E_g (theoretical) /eV	1.45[b]	1.01[b]	0.95[b]	0.96[a]	1.0[a]

[a]Ref.[4]; [b]Ref. [16]

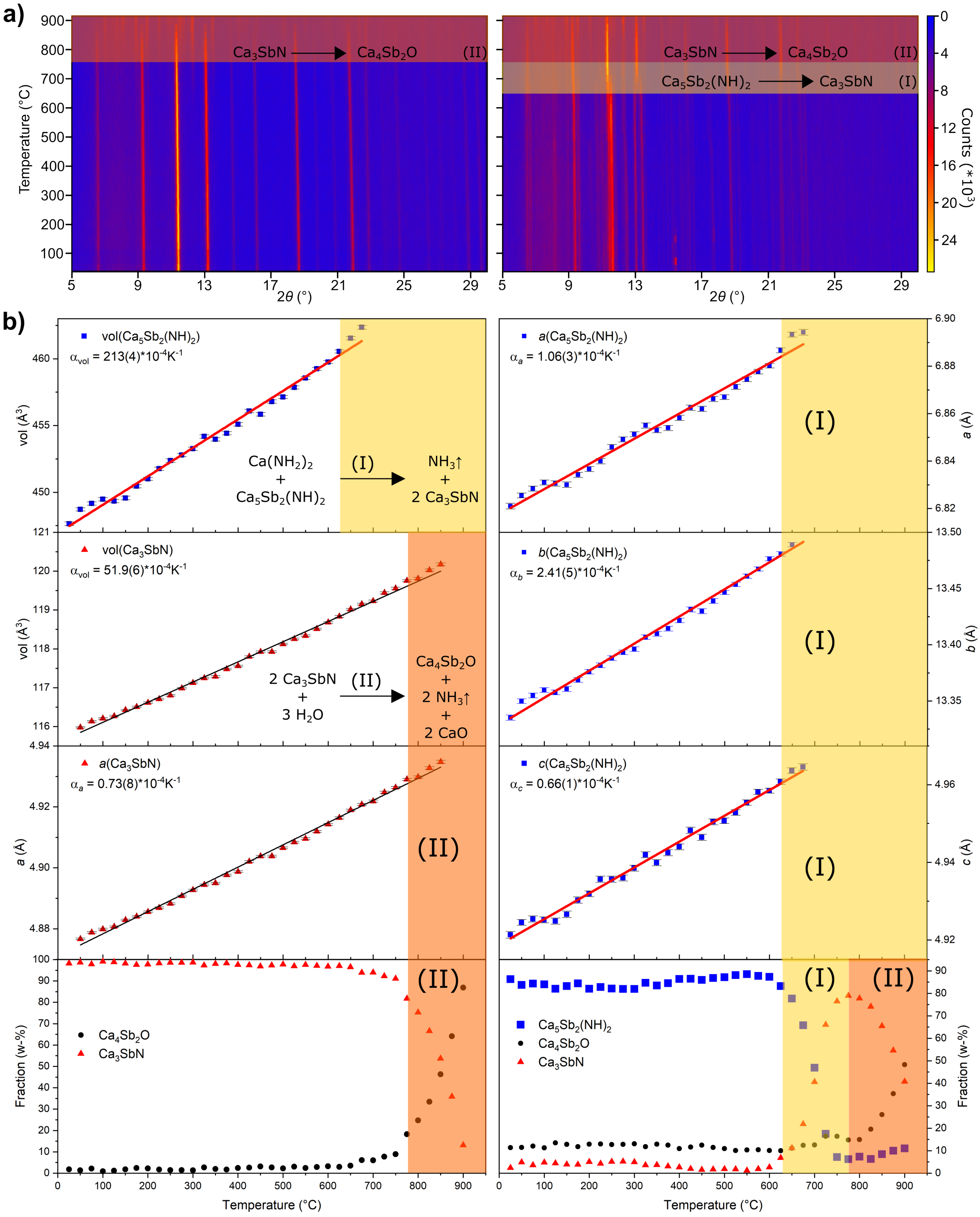

Figure S3 a) Temperature dependent powder X-ray diffraction data of Ca_3SbN and $Ca_5Sb_2(NH)_2$ (50K steps). **b)** Temperature-dependency of the unit cell parameters *a*, *b*, *c* and the volume. The linear thermal expansion was fitted with the thermal expansion coefficient *α* determined by the slope. Refined phase fractions of $Ca_5Sb_2(NH)_2$, Ca_4Sb_2O and Ca_3SbN are shown at the bottom and the observed reactions (I and II) are marked in yellow and orange. Standard uncertainties are shown by Black error bars.

2 Supplementary calculations

2.1 Oxygen impurities

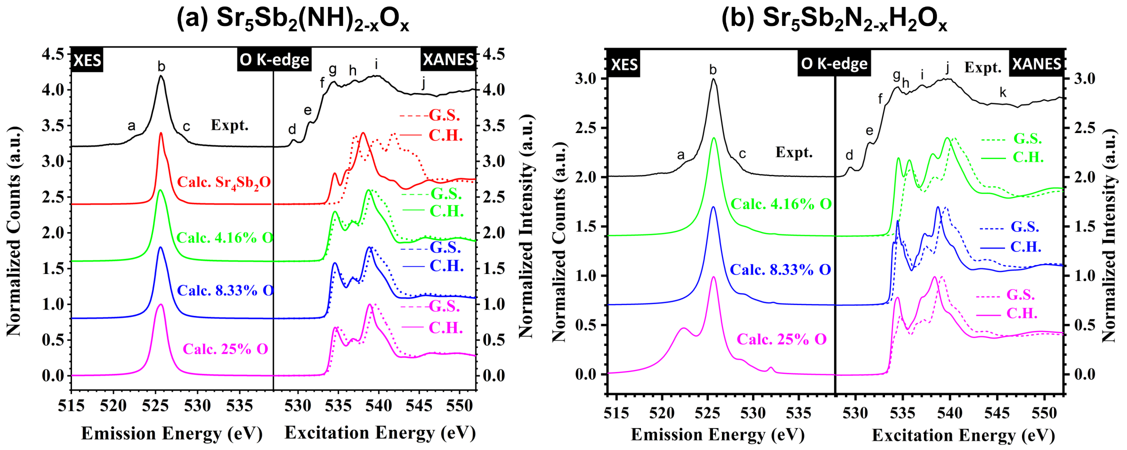

Figure S4 Experimental (black) and calculated O K-edge XES and XANES spectra of **(a)** $Sr_5Sb_2(NH)_{2-x}O_x$, and **(b)** $Sr_5Sb_2N_{2-x}H_2O_x$ with different concentrations of oxygen substitution, and secondary oxide phase (red) to identify the form of oxygen impurities. In the XANES spectra, dashed lines represent ground-state calculations, while solid lines indicate core-hole calculations.

As we cannot exclude a certain amount of oxygen incorporation from samples during synthesis, the question arises as to whether O is incorporated into the nominal structure or present in the form of a separate impurity phase. O K-edge XES and XANES spectra were measured for $Ca_5Bi_2(NH)_2$, $Ca_5Sb_2(NH)_2$, and $Sr_5Sb_2(NH)_2$ to evaluate the presence and nature of oxygen impurities. Calculations for secondary phase oxide impurities - Bi_2O_3, Ca_4Sb_2O, and Sr_4Sb_2O were performed that were suggested by our pXRD measurements of $Ca_5Bi_2(NH)_2$, $Ca_5Sb_2(NH)_2$, and $Sr_5Sb_2(NH)_2$.
To systematically investigate the presence of oxygen as oxonitride impurities in $Sr_5Sb_2(NH)_2$, a 2 × 1 × 3 supercell containing 24 symmetry-equivalent nitrogen atoms was constructed. Oxonitride impurities were then introduced by substituting one, two and six NH groups with oxygen, corresponding to oxygen concentrations of 4.16%, 8.33% and 25%, respectively (Figure S4a). We find that our approach is not sufficiently sensitive to discriminate between NH groups substituted by O, as the calculated O K-edge XES/XANES spectra remain unchanged with increasing oxygen concentration. In addition to this model, we also test a model in which the oxygen atoms substitute nitrogen atoms while hydrogen atoms remain in the structure (Figure S4b). Since O has a higher electronegativity than N, the O for N substitution results in an n-type semiconductor. We consider the first model (O substituting NH) chemically more reasonable. The spectral features *d* and *e* in the measured O K-edge XANES spectra of $Sr_5Sb_2(NH)_{2-x}O_x$, are likely associated with surface oxidation. Both defect models were also examined for the $Ca_5Bi_2(NH)_2$ and $Ca_5Sb_2(NH)_2$ samples, leading to the same conclusion. Overall, the results suggest that the measured spectra could arise from a combination of the secondary oxide phases and the defect oxide.

2.2 Density of States and Electron Density map

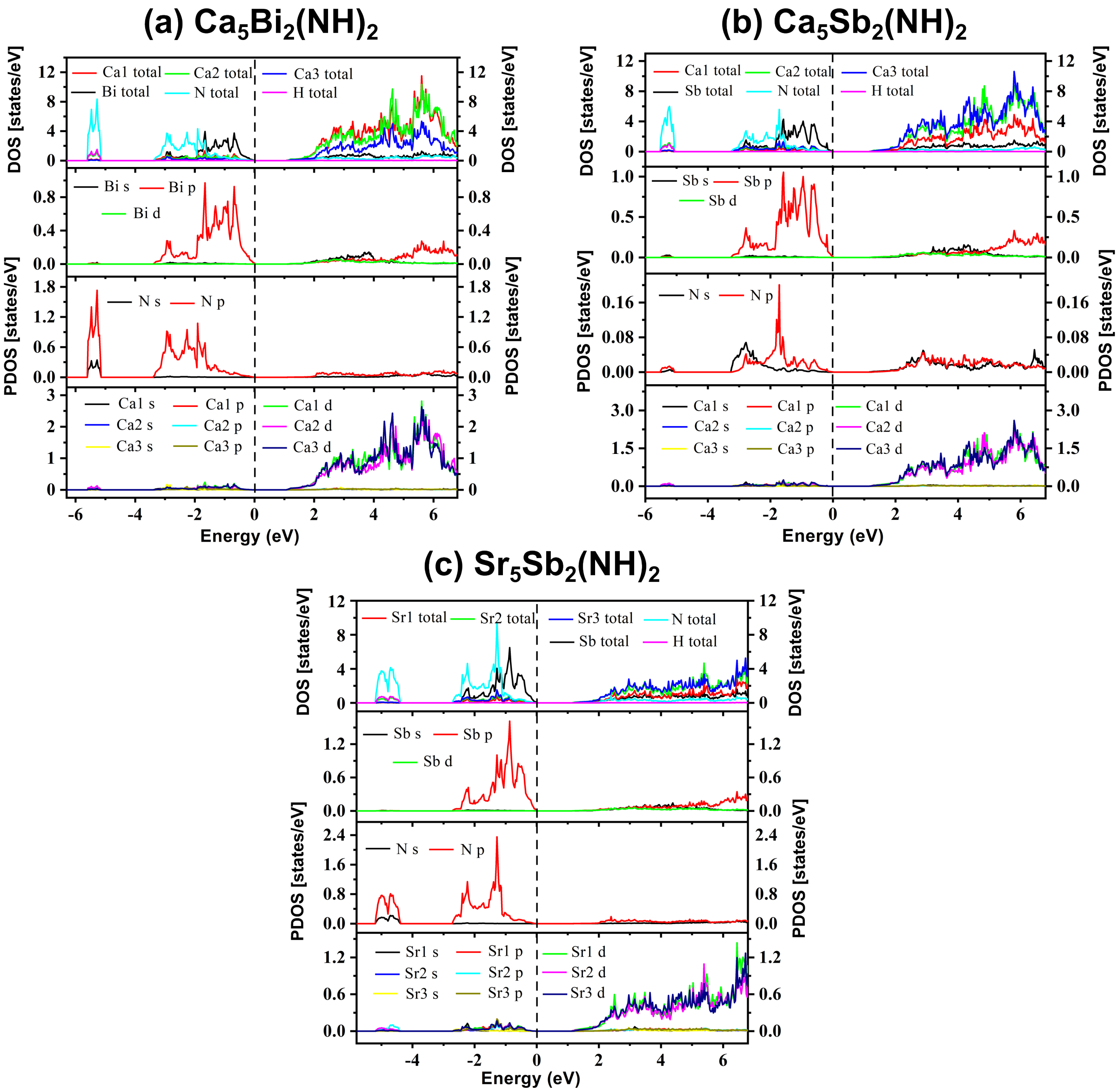

Figure S5 Calculated total density of states (DOS) per unit cell (top panels) and partial density of states (PDOS) per atom (bottom panels) for **a)** $Ca_5Bi_2(NH)_2$, **b)** $Ca_5Sb_2(NH)_2$, and **c)** $Sr_5Sb_2(NH)_2$ imide antiperovskite compounds, obtained using the PBE-GGA functional.

The density of states (DOS) and electron density plots were analyzed to gain deeper insight into the electronic structure of the $AE_5Pn_2(NH)_2$ compounds. Figure S5a-c shows the calculated total and partial DOS for each compound. Below the Fermi level (set at 0 eV), the valence band region consists of two distinct sub-bands. The lower energy sub-band originates from localized electronic states that do not significantly participate in chemical bonding. For both $Ca_5Bi_2(NH)_2$ and $Ca_5Sb_2(NH)_2$, the valence band is predominantly composed of occupied p-states of N and Bi/Sb atoms, while the conduction band is primarily formed by unoccupied d-states of Ca1, Ca2, and Ca3. In the case of $Sr_5Sb_2(NH)_2$, the valence band similarly originates from the p-states of N and Sb, whereas the conduction band is dominated by the d-states of all three inequivalent Sr atoms (Sr1, Sr2, and Sr3).

Now, the electron density map provides a detailed visual representation of the bonding interactions in the $Ca_5Sb_2(NH)_2$ structure, as illustrated in Figure S6. The electron density around the nitrogen atom is significantly higher than that around hydrogen, consistent with the formation of a polar covalent N–

H bond. The nearly spherical electron density contours around Ca2 and nitrogen exhibit slight overlap, suggesting a weak coordinate (dative) interaction, likely arising from lone pair donation by nitrogen to the electron-deficient Ca^{2+} atom. In contrast, only a very weak interaction is observed between Ca3 and the N–H group, while there is no observable interaction between Ca1 and N–H.

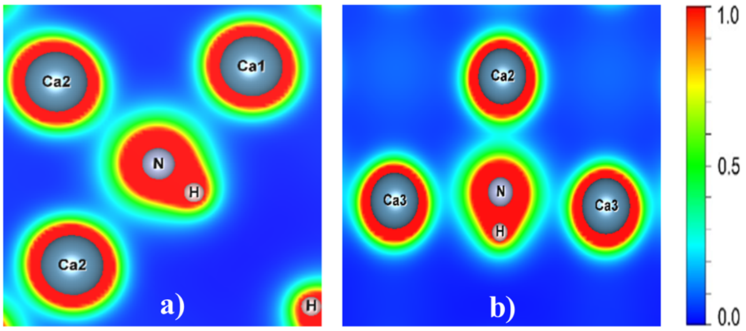

Figure S6 Calculated valence electron density maps of $Ca_5Sb_2(NH)_2$ in the (001) plane, to visualize the N–H and NH–Ca1/Ca2/Ca3 bonding environment, with minimum and maximum cutoff values of 0.001 and 0.8 e/Å^3, respectively.

2.3 Optical absorption spectra and SLME

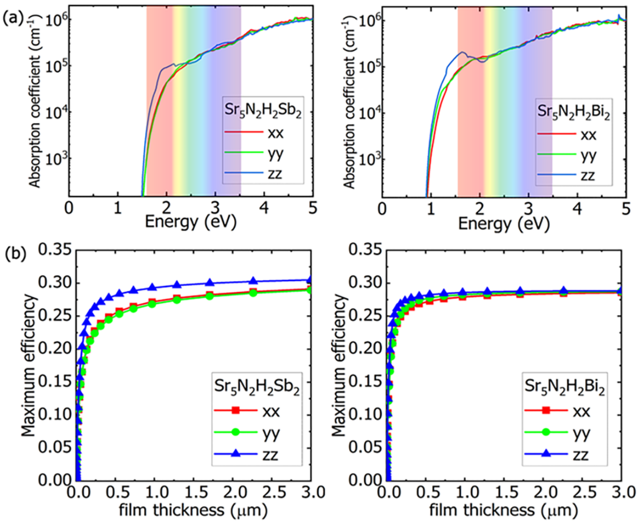

Figure S7 a) Optical absorption spectra and **b)** SLME as a function of film thickness of $Sr_5Sb_2(NH)_2$ and $Sr_5Bi_2(NH)_2$.

2.4 Effective mass of hole and electrons

Table S18: Effective mass of holes and electrons for $Ca_5As_2(NH)_2$, $Ca_5Sb_2(NH)_2$, $Sr_5Sb_2(NH)_2$ and $Sr_5Bi_2(NH)_2$.

	m_h^*/m_0			m_e^*/m_0		
	[100]	[010]	[001]	[100]	[010]	[001]
$Ca_5As_2(NH)_2$	0.506	0.574	1.103	0.711	1.020	0.405
$Ca_5Sb_2(NH)_2$	0.515	0.622	0.877	0.737	1.035	0.959
$Ca_5Bi_2(NH)_2$	0.399	0.488	0.752	0.782	1.072	0.922
$Sr_5Sb_2NH)_2$	0.556	0.446	1.019	0.708	0.841	0.750
$Sr_5Bi_2(NH)_2$	0.457	0.383	0.860	0.546	0.589	0.578

2.5 Band structures and densities of states of $Sr_5Bi_2(NH)_2$

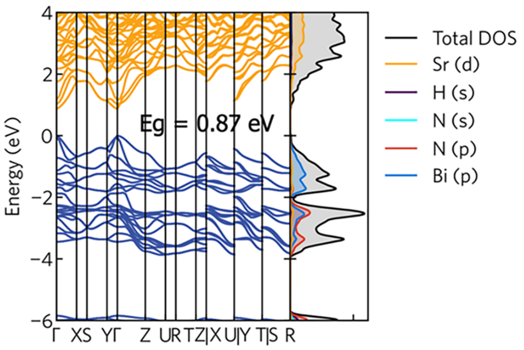

Figure S8 Band structures and densities of states of $Sr_5Bi_2(NH)_2$.

References

[52] J. Häusler, W. Schnick, *Chem. Eur. J.* **2018**, *24,* 11864–11879.
[53] *APEX3*, v2016.5-0, Bruker AXS Inc, Madison, USA, **2016**.
[54] G. M. Sheldrick, *XPREP,* Version 2008/2; Bruker AXS Inc., Madison, USA, **2008**.
[55] L. J. Farraugia, *J. Appl. Crystallogr.* **1999**, *32,* 837–838.
[56] G. M. Sheldrick, *Acta Cryst. A* **2015**, *71,* 3–8.
[57] C. B. Hübschle, G. M. Sheldrick, B. Dittric, *J. Appl. Crystallogr.* **2011**, *44,* 1281–1284.
[58] A. Coelho, *TOPAS Academics, Version 6,* Coelho Software, Brisbane,Australia, **2016**.
[59] P. Kubelka, F. Munk, *Z. Technol. Phys.* **1931**, *12,* 593–601.
[60] M. Boots, D. Muir, A. Moewes, *J. Synchrotron Rad.* **2013**, *20,* 272–285.
[61] G. Kresse, J. Furthmüller, *Phys. Rev. B* **1996**, *54,* 11169–11185.
[62] J. P. Perdew, K. Burke, M. Ernzerhof, *Phys. Rev. Lett.* **1996**, *77,* 3865–3868.
[63] J. Heyd, G. E. Scuseria, M. Ernzerhof, *J. Chem. Phys.* **2003**, *118,* 8207–8215.
[64] M.-H. Du, *J. Phys. Chem. Lett.* **2015**, *6,* 1461–1466.
[65] D. Han, M.-H. Du, C.-M. Dai, D. Sun, S. Chen, *J. Mater. Chem. A* **2017**, *5,* 6200–6210.
[66] A. M. Ganose, A. J. Jackson, D. O. Scanlon, *J. Open Source Softw.* **2018**, *3,* 717.
[67] L. Yu, A. Zunger, *Phys. Rev. Lett.* **2012**, *108,* 068701.

[68] P. Blaha, K. Schwarz, F. Tran, R. Laskowski, G. K. Madsen, L. D. Marks, *J. Chem. Phys.* **2020**, *152,* 074101.
[69] F. Tran, P. Blaha, *Phys. Rev. Lett.* **2009**, *102,* 226401.
[70] J. Camargo-Martínez, R. Baquero, *Phys. Rev. B* **2012**, *86,* 195106.
[71] K. Schwarz, A. Neckel, J. Nordgren, *J. Phys. F: Met. Phys.* **1979**, *9,* 2509.
[72] J. McLeod, R. Green, E. Kurmaev, N. Kumada, A. Belik, A. Moewes, *Phys. Rev. B* **2012**, *85,* 195201.
